# Cytokine signatures of end organ injury in COVID-19

**DOI:** 10.1038/s41598-021-91859-z

**Published:** 2021-06-15

**Authors:** Luis G. Gómez-Escobar, Katherine L. Hoffman, Justin J. Choi, Alain Borczuk, Steven Salvatore, Sergio L. Alvarez-Mulett, Manuel D. Galvan, Zhen Zhao, Sabrina E. Racine-Brzostek, He S. Yang, Heather W. Stout-Delgado, Mary E. Choi, Augustine M. K. Choi, Soo Jung Cho, Edward J. Schenck

**Affiliations:** 1 Division of Pulmonary and Critical Care Medicine, Weill Department of Medicine, Weill Cornell Medicine, New York, NY USA; 2 Division of Biostatistics, Department of Population Health Sciences, Weill Cornell Medicine, New York, NY USA; 3 Division of General Internal Medicine, Weill Department of Medicine, Weill Cornell Medicine, New York, NY USA; 4 Department of Pathology and Laboratory Medicine, Weill Cornell Medicine, New York, NY USA; 5 Advanced Diagnostics Complement Laboratory, National Jewish Health, Denver, CO USA; 6 Division of Nephrology and Hypertension, Weill Department of Medicine, Weill Cornell Medicine, New York, NY USA; 7 Department of Medicine, NewYork-Presbyterian Hospital/Weill Cornell Medicine, New York, NY USA

**Keywords:** Translational research, Infectious diseases

## Abstract

Increasing evidence has shown that Coronavirus disease 19 (COVID-19) severity is driven by a dysregulated immunologic response. We aimed to assess the differences in inflammatory cytokines in COVID-19 patients compared to contemporaneously hospitalized controls and then analyze the relationship between these cytokines and the development of Acute Respiratory Distress Syndrome (ARDS), Acute Kidney Injury (AKI) and mortality. In this cohort study of hospitalized patients, done between March third, 2020 and April first, 2020 at a quaternary referral center in New York City we included adult hospitalized patients with COVID-19 and negative controls. Serum specimens were obtained on the first, second, and third hospital day and cytokines were measured by Luminex. Autopsies of nine cohort patients were examined. We identified 90 COVID-19 patients and 51 controls. Analysis of 48 inflammatory cytokines revealed upregulation of macrophage induced chemokines, T-cell related interleukines and stromal cell producing cytokines in COVID-19 patients compared to the controls. Moreover, distinctive cytokine signatures predicted the development of ARDS, AKI and mortality in COVID-19 patients. Specifically, macrophage-associated cytokines predicted ARDS, T cell immunity related cytokines predicted AKI and mortality was associated with cytokines of activated immune pathways, of which IL-13 was universally correlated with ARDS, AKI and mortality. Histopathological examination of the autopsies showed diffuse alveolar damage with significant mononuclear inflammatory cell infiltration. Additionally, the kidneys demonstrated glomerular sclerosis, tubulointerstitial lymphocyte infiltration and cortical and medullary atrophy. These patterns of cytokine expression offer insight into the pathogenesis of COVID-19 disease, its severity, and subsequent lung and kidney injury suggesting more targeted treatment strategies.

## Introduction

Since the outbreak of severe acute respiratory syndrome coronavirus 2 (SARS-CoV-2) infection in December 2019, more than 25 million have developed Coronavirus disease 19 (COVID-19),with greater than 840,000 deaths^1^. Although patient characteristics vary by geographic location and pandemic stage, underlying conditions such as obesity, hypertension, chronic obstructive pulmonary disease, and diabetes mellitus are consistent risk factors for severe pneumonia^2–5^.

In addition to pneumonia, COVID-19 patients are at high risk of developing multiorgan systemic complications, including acute respiratory distress syndrome (ARDS), myocardial dysfunction, thrombosis, and acute kidney injury (AKI)^6, 7^. In COVID-19 patients who require hospitalization, ARDS occurs in 14% of patients and AKI occurs in 6–9%^8, 9^. In intensive care unit (ICU) cohorts, ARDS and AKI are even more common, affecting 73% and 43%, respectively^7^. These complications contribute to the high in-hospital mortality of COVID-19 patients. Although mortality rates vary by location, the latest data shows an overall in-hospital mortality rate of 10%^3, 7^.

Increasing evidence shows COVID-19 disease progression and severity may be driven by a dysregulated immunologic response due to over-activation of innate immune pathways, which results in the release of inflammatory cytokines and chemokines, and a corresponding depletion of several lymphocyte populations^10–14^. Overproduction of proinflammatory cytokines such as interleukin (IL)-1α, IL-1β, IL-6, IL-10, and tumor necrosis factor-α (TNF-α) have been described in multiple studies compared to healthy controls^15, 16^. Despite these reports, there is little data comparing the cytokine profiles of confirmed COVID-19 patients to control patients who present to a hospital in the same time period with symptoms closely resembling COVID-19 but a negative PCRtest. It is also unclear how cytokine expression correlates with clinical parameters and evolves early in the course of an admission to the hospital. In addition, it remains unknown whether specific patterns of dysregulated cytokines are associated with the development of distinct organ dysfunction such as ARDS and AKI in COVID-19. Identifying potentially diverging inflammatory pathobiology in specific organ dysfunction could suggest differential avenues of treatment. Therefore, the main objective of our study was to assess differences in inflammatory cytokines in COVID-19 patients compared to contemporaneously hospitalized controls, and then to analyze the relationship between these cytokines and the development of mortality, ARDS and AKI.

## Results

### Demographic and baseline characteristics

A sample of 141 patients were included; 90 patients had confirmed COVID-19 and 51 were controls. A total of 141 day 1 specimen, 63 day 2 and 63 day 3 samples were collected. Control patients most commonly presented with bacterial pneumonia and other respiratory tract infections, Supplementary Table 1. Demographic and baseline characteristics between COVID-19 and control patients are shown in Table 1. The median age of the COVID-19 and control groups were similar 66 [57–77] versus 64 [57–78]; p = 0.50. The COVID-19 group included 37% females compared to 55% in the control group; p = 0.054. The COVID-19 group had a higher body mass index (BMI) 27.6 [23.2–31.1] versus 25.2 [21.7–28.8]; p = 0.030, but were less likely to have active cancer, immunosuppression, and were less likely to be active smokers compared to the controls. Additionally, the COVID-19 group had a higher day 1 of hospital admission temperature, respiratory rate, and heart rate compared to the control group.

**Table 1:** Demographics and baseline characteristics of COVID-19 patients and controls.

Characteristic	Controls N = 51	COVID-19 N = 90	p-value
**Demographics**			
Age (median, IQR)	64 (57, 78)	66 (57, 77)	0.5
Sex, female (n, %)	28 (55%)	33 (37%)	0.054
**Race (n, %)**			﻿< 0.001
Asian	3 (5.9%)	6 (7.5%)	
Black	8 (16%)	12 (15%)	
Other	3 (5.9%)	18 (22%)	
White	29 (57%)	44 (55%)	
BMI (median, IQR)	25.2 (21.7, 28.8)	27.6 (23.2, 31·1)	0.030
**Smoking status (n, %)**			0.﻿0﻿2﻿9
Active smoker	4 (8.0%)	0 (0%)	
Former smoker	14 (28%)	30 (33%)	
Never smoker	32 (64%)	60 (67%)	
**Comorbidities (n, %)**			
CAD	12 (24%)	13 (14%)	0.3
DM	9 (18%)	28 (31%)	0.12
HTN	27 (53%)	51 (57%)	0.8
CVA	9 (18%)	9 (10%)	0.3
CKD/ESRD	6 (12%)	9 (10%)	> 0.9
Cirrhosis	1 (2.0%)	1 (1.1%)	> 0.9
COPD	7 (14%)	6 (6.7%)	0.2
Asthma	6 (12%)	10 (11%)	> 0.9
Active cancer	19 (37%)	11 (12%)	﻿0.﻿0﻿0﻿1
Immunosuppressed state^a^	18 (35%)	4 (4.4%)	< ﻿0﻿.00﻿1
**Home medications (n, %)**			
Immunosuppressive medications (past 30 days)	16 (31%)	15 (17%)	0﻿.0﻿70
ACE/ARBs	13 (25%)	25 (28%)	> 0.9
Statins	22 (43%)	33 (37%)	0.6
NSAIDs	16 (31%)	24 (27%)	0.7
PPIs	15 (29%)	21 (23%)	0.6
**Day 1 vital signs (median, IQR)**			
Highest temperature (°C)	37.00 (36.75, 37.70)	38.30 (37.50, 39.00)	﻿< ﻿0﻿.﻿0﻿0﻿1
Highest heart rate	95 (86, 110)	100 (91, 105)	0.2
Highest respiratory rate	20.0 (18.0, 22.0)	22.0 (20.0, 30.0)	< ﻿0﻿.﻿0﻿0﻿1
Lowest systolic blood pressure	106 (100, 118)	105 (92, 113)	0.2

*BMI* Body Mass Index, *CAD* coronary artery disease, *DM* diabetes mellitus, *HTN* hypertension, *CVA* cerebrovascular accident, *CKD/ESRD* chronic kidney disease/end stage renal disease, *COPD* chronic obstructive pulmonary disease, *ACE/ARBs* angiotensin-converting enzyme inhibitors/angiotensin-II receptor blockers, *NSAIDs* nonsteroidal anti-inflammatory drugs, *PPIs* proton pump inhibitors.

^a^Chemotherapy or radiotherapy within last 6 months; inherited immunodeficiency.

### Clinical laboratory characteristics

Baseline clinical laboratory values stratified by the COVID-19 and control groups are provided in Table 2. There were several between group differences in clinical laboratory tests on admission. Specifically, COVID-19 patients had lower absolute lymphocyte counts (0.80 [0.5–1.1] vs 1.08 [0.55, 1.79]; p = 0.032), and platelet counts (173 [136–227] vs 224 [148, 270]; p = 0.040), but higher levels of hemoglobin (12.50 [10.70, 13.40] vs 11 [9.75–13.25]; p = 0.041) compared to controls. In serum chemistries COVID-19 patients had lower albumin level (2.80 [2.40–3.23] vs 3.50 [2.90, 3.90]; p < 0.001), alanine aminotransferase (ALT) (20 [14, 37] vs 30 [19–45]; p < 0.039), aspartate aminotransferase(AST) (24 [19, 34] vs 39 [27–69]; p < 0.001) and lactate (0.96 [0.76–1.25] vs 1.30 [1.00, 1.70]; p < 0.001). Other laboratory results were similar between groups (Table 2).

**Table 2:** Initial laboratory results within 72 h of admission of COVID-19 patients and controls.

Laboratory result	Controls N = 51	COVID-19 N = 90	p-value
**Routine blood**			
White blood cell, 10^3^ µL	7.2 (4.8, 10.7)	5.8 (4.3, 7.9)	0.13
Neutrophils, 10^3^ µL	4.5 (2.9, 6.8)	4.0 (3.1, 6.6)	0.9
Lymphocytes, 10^3^ µL	1.08 (0.55, 1.79)	0.80 (0.50, 1.10)	0﻿.﻿0﻿3﻿2
Platelet, 10^3^ µL	224 (148, 270)	173 (136, 227)	﻿0.﻿04﻿0
Hemoglobin, g/dL	11.00 (9.75, 13.25)	12.50 (10.70, 13.40)	﻿0﻿.﻿0﻿4﻿1
Neutrophil/lymphocytes ratio	5.2 (2.1, 7.6)	5.5 (3.1, 10.1)	0.067
**Coagulation function**			
Partial thromboplastin time, s	31.0 (27.1, 33.8)	32.6 (29.8, 34.6)	0.2
Prothrombin time, s	13.05 (11.65, 14.93)	13.85 (12.80, 15.15)	0.2
d-dimer, ng/mL	242 (150, 444)	389 (234, 698)	0.14
Fibrinogen, mg/dL	375 (221, 417)	488 (383, 573)	0.15
**Blood biochemistry**			
Albumin, g/dL	3.50 (2.90, 3.90)	2.80 (2.40, 3.23)	﻿< 0.001
Alanine aminotransferase, U/L	20 (14, 37)	30 (19, 45)	﻿0﻿.﻿0﻿3﻿9
Aspartate aminotransferase, U/L	24 (19, 34)	39 (27, 69)	< ﻿0﻿.0﻿0﻿1
Total bilirubin, mg/dL	0.50 (0.40, 0.90)	0.60 (0.40, 0.85)	> 0.9
Creatinine, mg/dL	0.93 (0.69, 1.21)	0.94 (0.76, 1.33)	0.6
Ferritin, ng/dL	386 (103, 1167)	749 (381, 1383)	0.3
Lactate, mmol/L	1.30 (1.00, 1.70)	0.96 (0.76, 1.25)	﻿0﻿.﻿010
Glucose, mg/dL	108 (92, 134)	106 (93, 133)	0.6
**Inflammatory markers**			
C-reactive protein, µg/mL	4 (2, 7)	10 (5, 16)	0.10
Erythrocyte sedimentation rate, mm/h	60 (46, 60)	55 (20, 71)	> 0.9
Procalcitonin, ng/mL	0.24 (0.13, 0.46)	0.14 (0.07, 0.36)	0.3
**Arterial blood gas analysis**			
pH	7.60 (7.60, 7.60)	7.40 (7.31, 7.44)	0.092
PaO_2_, mmHg	78 (78, 78)	74 (55, 84)	0.8
PaCO_2_, mmHg	44 (44, 44)	38 (33, 49)	0.7

### Initial organ failure, respiratory support and clinical outcomes

Differences in baseline severity of illness were evaluated using admission burden of organ failure, patterns of chest imaging and initial level of oxygen. Despite requiring similar overall levels of supplemental oxygen at admission (p = 0.2, Table 3), 47% of the COVID-19 group were treated with any oxygen compared to 29% of the control population. Day 1 of hospital admission SOFA scores were higher in COVID-19 patients when compared to controls (2.0 [1.0, 5.0] vs 1.0 [0.0–3.0]; p = 0.001). Additionally, chest X-ray findings upon arrival to the emergency room were different between groups. The majority of COVID-19 patients had bilateral infiltrates at admission compared to controls (64% vs 13%; p < 0.001).

**Table 3:** Severity of Illness and outcomes of COVID-19 patients and controls.

Clinical status	Controls N = 51	COVID-19 N = 90	p-value
**Initial respiratory status**			
Highest level of supplemental oxygen in the first 3 h			0.2
HFNC/NRB/NIV mechanical ventilation	2 (3.9%)	8 (8.9%)	
Mechanical ventilation	2 (3.9%)	4 (4.4%)	
Nasal cannula	11 (22%)	30 (33%)	
None	36 (71%)	48 (53%)	
**CXR results at admission**			< 0﻿.﻿001
Bilateral infiltrates	6 (13%)	58 (64%)	
Clear	26 (58%)	10 (11%)	
Pleural effusion	0 (0%)	4 (4.4%)	
Unilateral infiltrates	6 (13%)	0 (0%)	
**Day 1 severity of illness (median, IQR)**			
SOFA score	1.0 (0.0, 3.0)	2.0 (1.0, 5.0)	﻿0﻿.﻿00﻿1
**Complications and clinical outcomes**			
Thromboembolic event	2 (3.9%)	11 (12%)	0.13
Respiratory co-infection	16 (31%)	20 (22%)	0.3
Acute respiratory distress syndrome (ARDS) requiring intubation	0 (0%)	36 (40%)	﻿< ﻿0.﻿00﻿1
**Acute kidney injury (AKI) stage**^**a**^			﻿< ﻿0.0﻿0﻿1
0	48 (94%)	49 (60%)	
1	2 (3.9%)	11 (14%)	
2	1 (2.0%)	5 (6.2%)	
3	0 (0%)	16 (20%)	
In-hospital mortality	5 (9.8%)	19 (21%)	0.14

AKI was defined according to KDIGO guidelines^21^.

ARDS was defined according to the Berlin Definition^22^.

*HFNC* High-flow nasal cannula, *NRB* non-rebreather mask, *NIV* non-invasive, *CXR* chest X-ray, *SOFA* sequential organ failure assessment.

^a^Patients with end stage renal disease who were on dialysis prior to admission were excluded.

To evaluate the relative in-patient morbidity and mortality, we followed the COVID-19 and control patients through their index hospitalization and documented incident complications to compare differences between the groups. COVID-19 patients more commonly developed ARDS (40% vs 0%; p < 0.001) as well as any kidney injury as shown in Table 3, including treatment with kidney replacement therapy (KRT) (14% vs 2%, p = 0.037) compared to controls. The 28-day and in-hospital mortality in the COVID-19 compared to the control groups were 19% vs 8% p = 0.13 and 21% vs 9.8%; p = 0.14 respectively.

### Inflammatory cytokine expression in COVID-19 compared to controls

**Figure 1:**
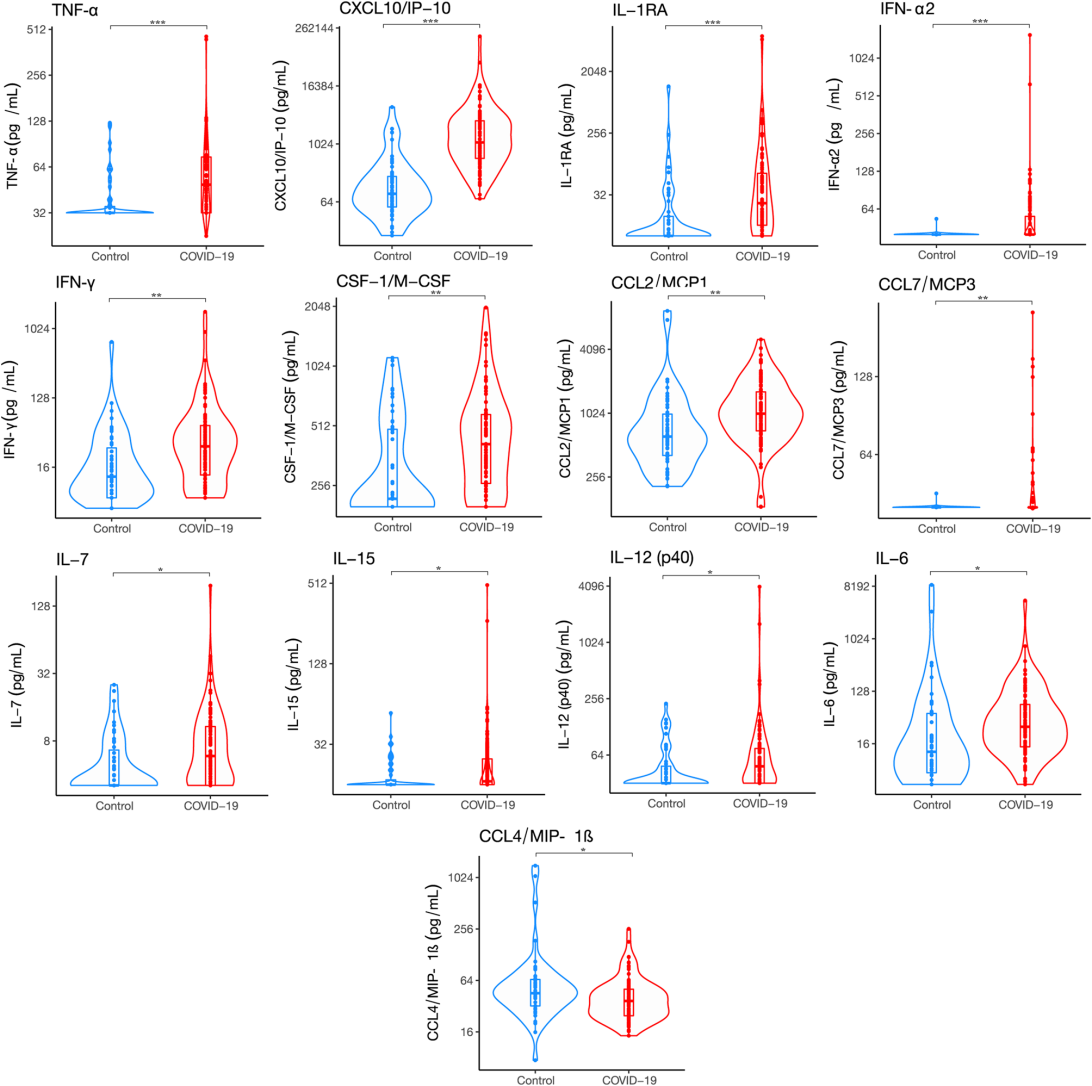
Cytokine expression of COVID19 and control patients. The curved line of the violin box plots show the density of day 1 of hospital admission cytokine expression levels. The horizontal line in the inner box plot represents the median and interquartile range. Each dot represents a subject (COVID19, n = 90; Control, n = 51). Significance of comparisons were determined by an unadjusted linear regression models using log-scaled cytokines and robust standard errors. P-values after adjustment for multiple comparisons accompany the respective comparisons. *p < 0.05, **p < 0.01, ***p < 0.001.

**Figure 2:**
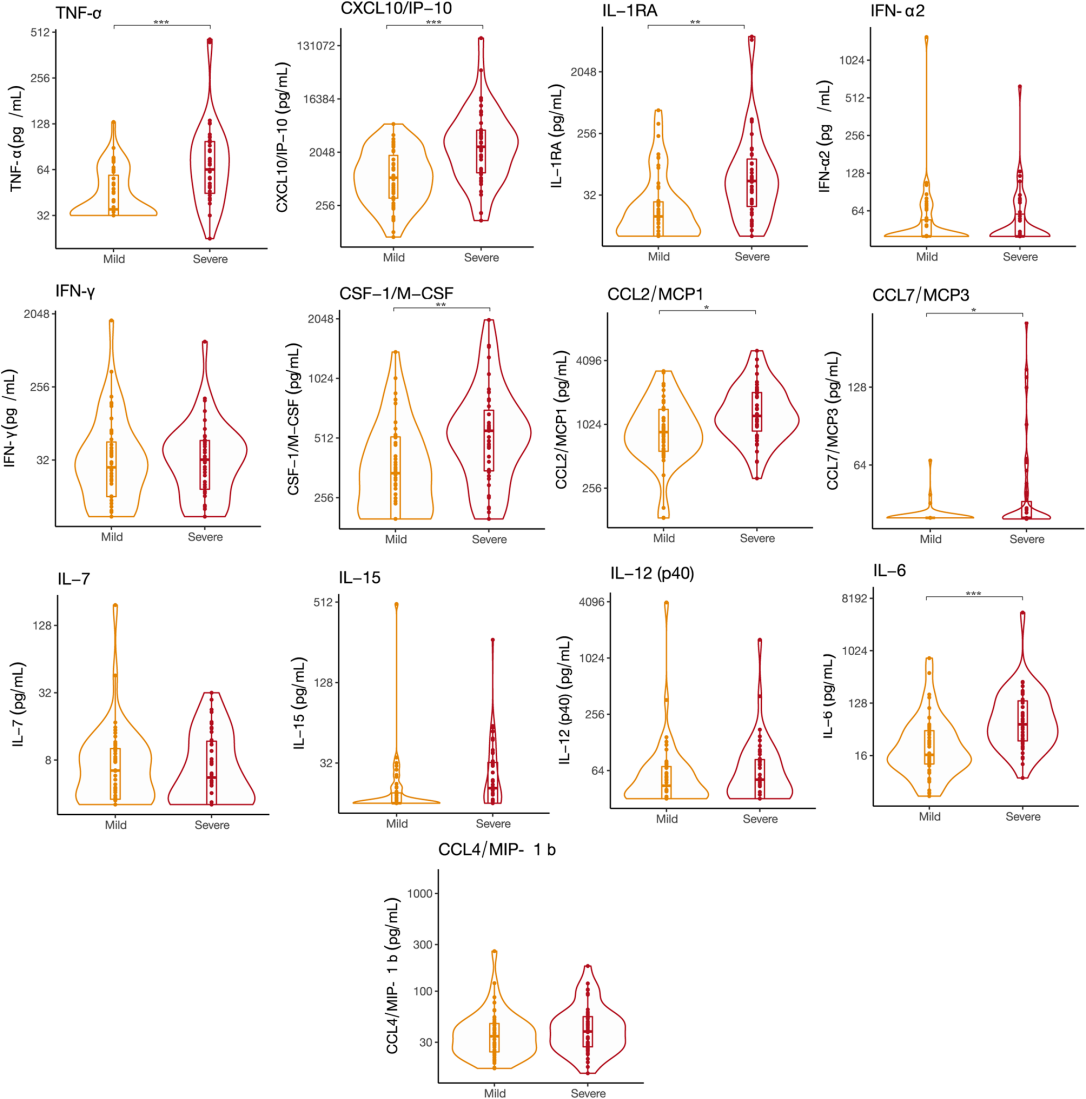
Cytokine expression of inflammatory cytokines in mild and severe COVID19 patients. The curved line of the violin box plots show the density of day 1 of hospital admission the cytokine expression levels. The horizontal line in the inner box plot represents the median and interquartile range. Each dot represents a subject (COVID-19, Mild = 56; Severe, n = 34). Significance of comparisons were determined by an unadjusted linear regression models using log-scaled cytokines and robust standard errors. p-values after adjustment for multiple comparisons accompany the respective comparisons. *p < 0.05, **p < 0.01, ***p < 0.001.

Our data thus far demonstrated that compared to controls, the baseline severity of illness was higher in the COVID-19 group and that they frequently developed in-patient complications. To explore whether these findings were related to differences in inflammatory cytokine expression, we analyzed the day 1 serum cytokine profile by 48plex. Several differences between the COVID-19 and control day 1 of hospital admission cytokine expression levels were identified. Specifically, there was a significant overexpression of IP-10, TNF-α, IFN-α2, IFN-γ, IL-1RA, MCP-3, M-CSF, IL-7, MCP-1, MIP-1β, IL-15, IL-12 (p40), PDGF AA, IL-6, FLT 3L, and IL-10 in COVID-19 patients, as shown in Fig. 1. The log_2_ fold-change differences between groups are shown in Supplementary Table 5. To expand these findings and identify whether there was dose–response relationship between overexpressed cytokines and the severity of COVID-19 pneumonia by the WHO classification, we examined whether cytokine expression levels associated with disease severity. When compared to mild disease, patients that developed severe pneumonia had a significant increase in IL1-RA, IL-6, IP-10, MCP-1, MCP-3, M-CSF, and TNF-α (Fig. 2).

### Cytokine correlation with clinical laboratory findings

**Figure 3:**
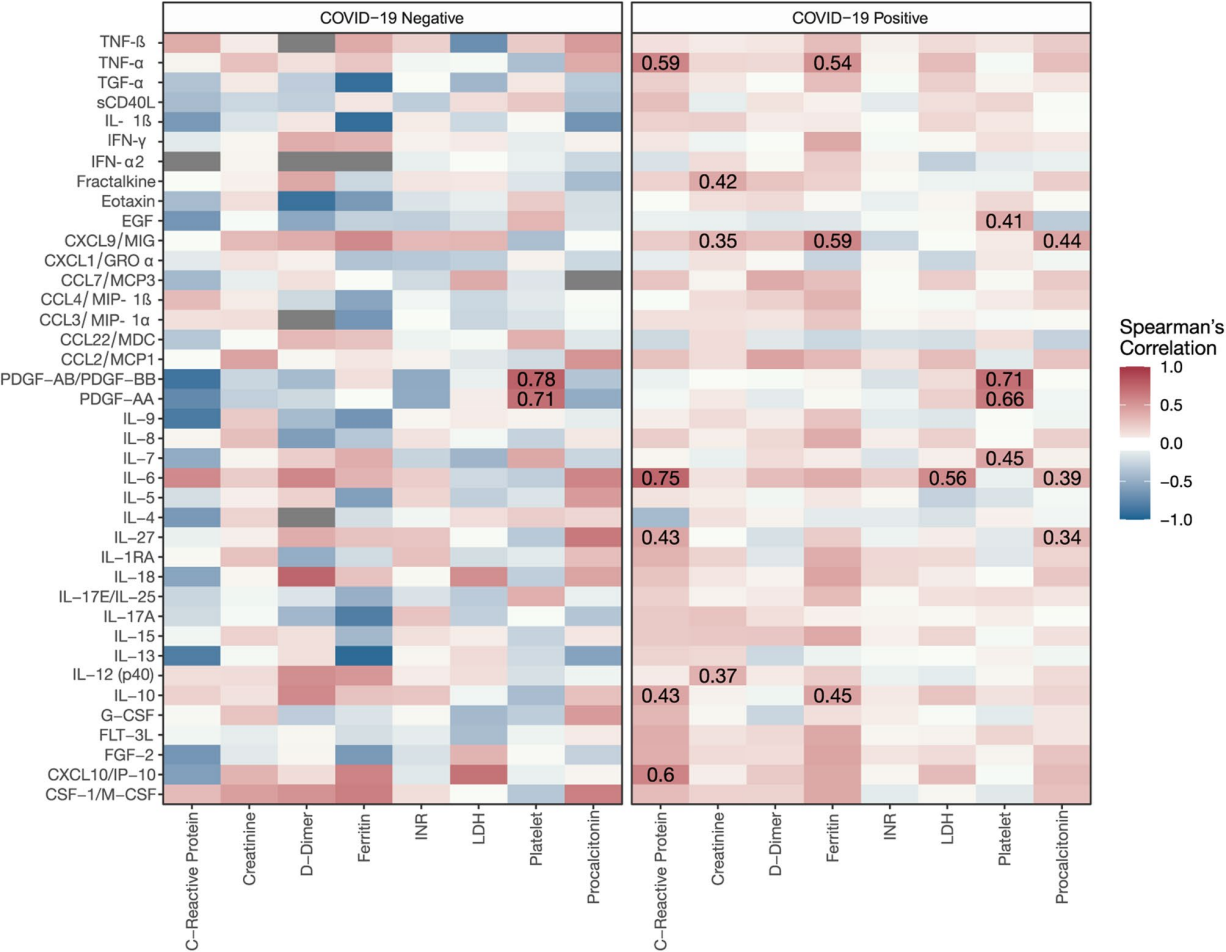
Cytokine and clinical laboratory correlations of COVID-19 and control patients. Correlation heatmap of 39 cytokines from patient serum comparing cytokine concentrations at day 1 of hospital admission with first clinical laboratory parameters obtained in the first 72 h of admission. Correlation heatmaps are stratified by COVID-19 patients and controls. Only significant correlations (p < 0.05) after adjustment for multiple comparisons are presented with a Spearman's correlation coefficient value. The Spearman's correlation coefficient is visualized by color intensity. *INR* International Normalized Ratio, *LDH* lactate dehydrogenase.

We next explored the relationship between the 48plex cytokines and routinely measured clinical laboratory values to understand how closely the baseline clinical and inflammatory phenotypes were correlated within COVID-19 and control patients. As shown in Fig. 3, clinical laboratory values were not associated with any inflammatory cytokines in the control population, with the exception of platelet levels positively correlated with PDGF-AB/BB (r = 0.78, p = 0.004) and PDGF-AA (r = 0.71, p < 0.001). For COVID-19 patients, multiple cytokines were correlated with clinical laboratory biomarkers. C-reactive protein (CRP) positively correlated with IL-6 expression (r = 0.75, p < 0.001), IP-10 (r = 0.6, p < 0.001), TNF-α (r = 0.59, p < 0.001), IL-27 (r = 0.43, p = 0.03), and IL-10 (r = 0.43, p = 0.03). Serum creatinine levels positively correlated with fractalkine (r = 0.42, p = 0.003), IL-12 (p40) (r = 0.37, p = 0.01), and monokine induced by IFN-γ (MIG) (r = 0.35, p = 0.02) (Fig. 3). In addition, there were significant correlations between ferritin levels and the expression of MIG (r = 0.59, p = 0.001), TNF-α (r = 0.54, p = 0.005), and IL-10 (r = 0.45, p = 0.046) (Fig. 3). Platelet counts were positively correlated with PDGF-AB/BB (r = 0.71, p < 0.001), PDGF-AA (r = 0.66, p < 0.001), IL-7 (r = 0.45, p < 0.001), and EGF (r = 0.41, p = 0.003) (Fig. 3). There were also significant correlations with procalcitonin and MIG (r = 0.44, p = 0.003), IL-6 (r = 0.39, p = 0.01), and IL-27 (r = 0.34, p = 0.046) (Fig. 3). In this population, D-Dimers and the International Normalized Ratio (INR) were not significantly correlated with any cytokine.

### Cytokine correlation with baseline organ dysfunction and other cytokines

**Figure 4:**
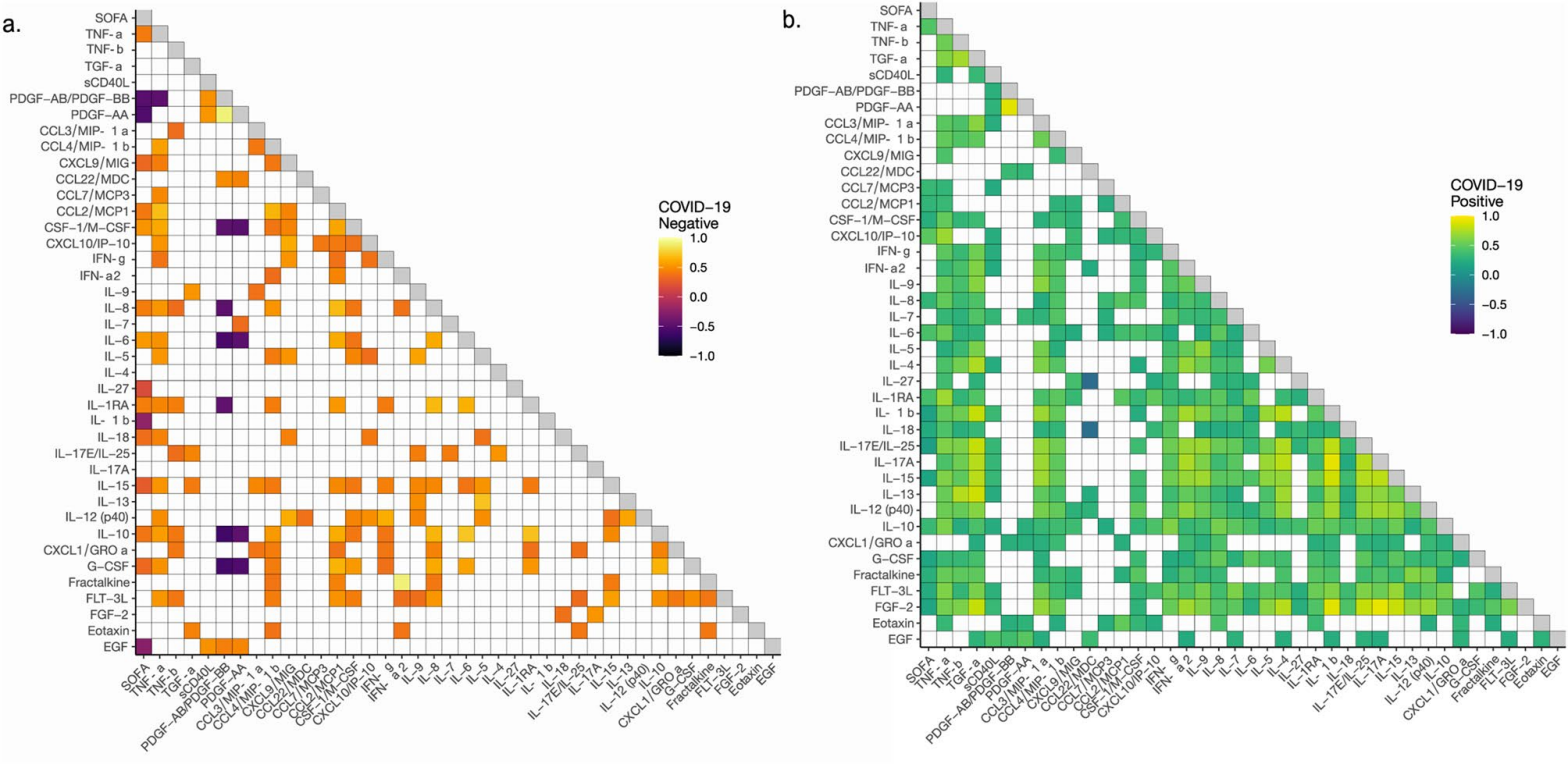
Cytokine and SOFA score correlations between positive and negative COVID-19 patients. Correlation matrix of 39 cytokines from patient serum comparing cytokine concentrations at day 1 of hospital admission with SOFA scores. Correlation heatmaps are stratified by COVID-19 patients and controls. Only significant correlations (p < 0.05) after adjustment for multiple comparisons are presented with a Spearman's correlation coefficient value. The Spearman's correlation coefficient is visualized by color intensity. *SOFA* Sequential Organ Failure Assessment.

We next examined the correlation between cytokine levels and admission SOFA score among COVID-19 and control patients to explore relationships with common clinical phenotypes of acute organ dysfunction. As illustrated in Fig. 4 Supplementary Fig. 3, there were differential correlations between cytokine levels and SOFA scores in the two groups. GCSF, IL-1RA, IL-6, IL-8, IL-10, IL-12 (p40), MCP-1, M-CSF, and TNF-α were correlated with SOFA scores in COVID-19 patients and controls, with increased cytokine expression positively correlating with higher SOFA scores (Fig. 4, Supplementary Fig. 4). Similarly, FGF-2, FLT-3L, IL-9, IL-17E/IL-25, IP-10, MCP-3, and MIP-1β were also positively correlated with SOFA scores in COVID-19 patients (Fig. 4, Supplementary Fig. 3). In control patients, IL-15, IL-18, IL-27, and CXCL9/MIG positively correlated with SOFA scores (Fig. 4, Supplementary Fig. 3). Interestingly, IL-1β was positively correlated with SOFA scores in COVID-19 patients, but remained negatively correlated in controls (Fig. 4, Supplementary Fig. 3).

Given the differential relationship between cytokines and clinical parameters in COVID-19 patients and controls we further analyzed the relationship between the cytokines in each group (Fig. 4). There were more cytokine to cytokine correlations in the COVID-19 group compared to controls. Additionally, distinguishable inflammatory cytokines and chemokines were positively correlated within COVID-19 patients, such as: macrophage induced chemokines (IL-1β, IL-1RA, IL-6, IL-12, IL-18, CXCL1/GROα, CCL7/MCP3, CCL2/MDC, CCL3/MIP-1α, TGF-α, TNF-β and IFN-α2), T-cell related interleukines (IL-4, IL-5, IL-13, IL-15, IL-17A, IL-17E/IL-25 and sCD40L) and stromal cell producing cytokine (IL-7).

### Cytokine correlation with clinical outcomes

**Figure 5:**
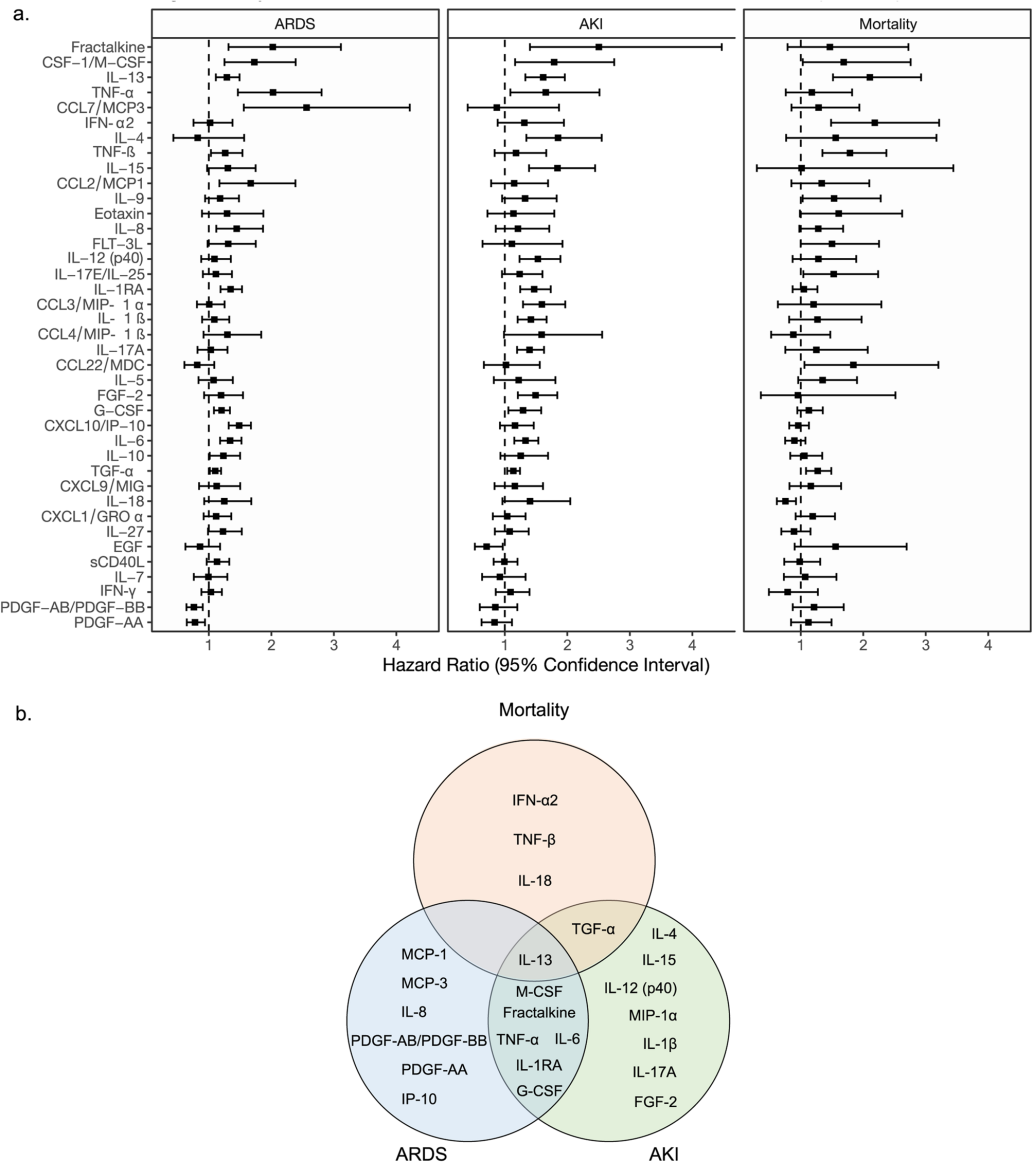
Associations between cytokine expression levels and clinical outcomes within COVID19 patients. (**a**) Forest plots representing the estimates of association for day 1 of hospital admission cytokine expression with clinical outcomes of mortality, need for intubation (ARDS), and development of acute kidney injury (AKI) among COVID-19 patients. Each box shows the estimated Hazard Ratio (HR) and each whisker represents the 95% Confidence Interval (CI) of the HR. Cox Proportional Hazard (PH) models with robust standard errors were used to compute all estimates with time 0 as day 1 of hospital admission. (**b**) Venn diagram showing 23 cytokines significantly associated (p < 0.05) with clinical outcomes such as mortality, ARDS, and development of AKI after pvalue adjustment for multiple comparisons.

Our data demonstrated a differential inflammatory phenotype in COVID-19 compared to controls with unique relationships between baseline cytokines, clinical labs, and organ dysfunction. We next evaluated whether differential baseline cytokine levels in the COVID-19 group associated with the development of subsequent clinical outcomes of ARDS, AKI and mortality. In the COVID-19 cohort, thirty-six patients developed ARDS (40%) (Table 3). The Cox Proportional Hazard (PH) regression model results for cytokines associated with ARDS within COVID-19 patients are shown in Fig. 5 and Supplementary Table 8c. In total, 13 cytokines were associated with ARDS (Fig. 5a, Supplementary Table 8c). The most relevant cytokines that were significantly associated with ARDS were MCP-3 (HR 2.56; 95% CI 1.56, 4.22; p = 0.002) TNF-α (HR 2.03; 95% CI 1.46, 2.80; p < 0.001), fractalkine (HR 2.02; 95% CI 1.31, 3.1; p = 0.006), M-CSF (HR 1.73; 95% CI 1.25, 2.39; p = 0.004), and MCP-1 (HR 1.67; 95% CI 1.17, 2.39; p = 0.02) (estimates for other significant cytokines are shown in Supplementary Table 8c).

Among eighty COVID-19 patients without end stage renal disease (ESRD), 32 (43.6%) developed AKI during their hospital stay (Table 3). The maximum AKI staging developed during hospitalization for COVID-19 patients was 13% for stage 1 AKI, 5.6% for stage 2 AKI, and 25% for Stage 3 AKI (Table 3). As shown in Supplementary Fig. 4, there was a significant association with IL-6 levels and AKI staging (p = 0.009) and relevant trends with TNF-α, IL-1RA, and FGF-2. The Cox PH regression model results for development of AKI within COVID-19 patients are shown in Fig. 5 and Supplementary Table 6a. In total, 15 cytokines were associated with the development of AKI. Within these cytokines, the most relevant were fractalkine (HR 2.50; 95% CI 1.40, 4.47; p = 0.007), IL-4 (HR 1.85; 95% CI 1.35, 2.55; p < 0.001), IL-15 (HR 1.84; 95% CI 1.39, 2.45; p < 0.001), M-CSF (HR 1.79; 95% CI 1.17, 2.75; p < 0.001), and TNF-α (HR 1.66; 95% CI 1.09, 2.52; p < 0.001) (Supplementary Table 8a).

Of the cohort, nineteen COVID-19 patients died (21%) during their hospital stay (Table 3). The adjusted Cox Proportional Hazard (PH) regression model results for cytokines associated with mortality within COVID-19 patients are displayed in Fig. 5 and Supplementary Table 8b. In total, 5 cytokines were associated with mortality. Specifically, the most relevant cytokines that were significantly associated with mortalityin COVID-19 patients are: IFN-β (HR 2.18; 95% CI 1.49, 3.21; p < 0.001), IL-13 (HR 2.11; 95% CI 1.52, 2.93; p < 0.001), TNF-β (HR 1.79; 95% CI 1.35, 2.37; p < 0.001), TGF-α (HR 1.27; 95% CI 1.09, 1.49; p = 0.03), and IL-18 (HR 0.76; 95% CI 0.62, 0.92; p = 0.049) (Supplementary Table 8b). Of note, IL-13, secreted by activated Th2 cells, constituting a counter-regulatory system for the inflammatory response was not only correlated mortality but also predictive of ARDS and AKI.

### Longitudinal changes in cytokine profile in the COVID-19 group

Potential longitudinal changes of cytokine levels throughout early days of the hospital were explored in 15 COVID-19 patients with cytokine measures for each of the first three days following admission to the general ward floor. There were no detectable trends for any of the cytokines over this time period (Supplementary Fig. 2, and Supplementary Tables 1 and 2). We validated this result using an 8-plex assay in a subset of 54 COVID-19 patients (Supplementary Table 4). Consistent with the findings in 48-plex, there were no detectable differences in cytokine expression within 3 days post admission.

### Histopathology findings of COVID-19 lung and kidney

**Figure 6:**
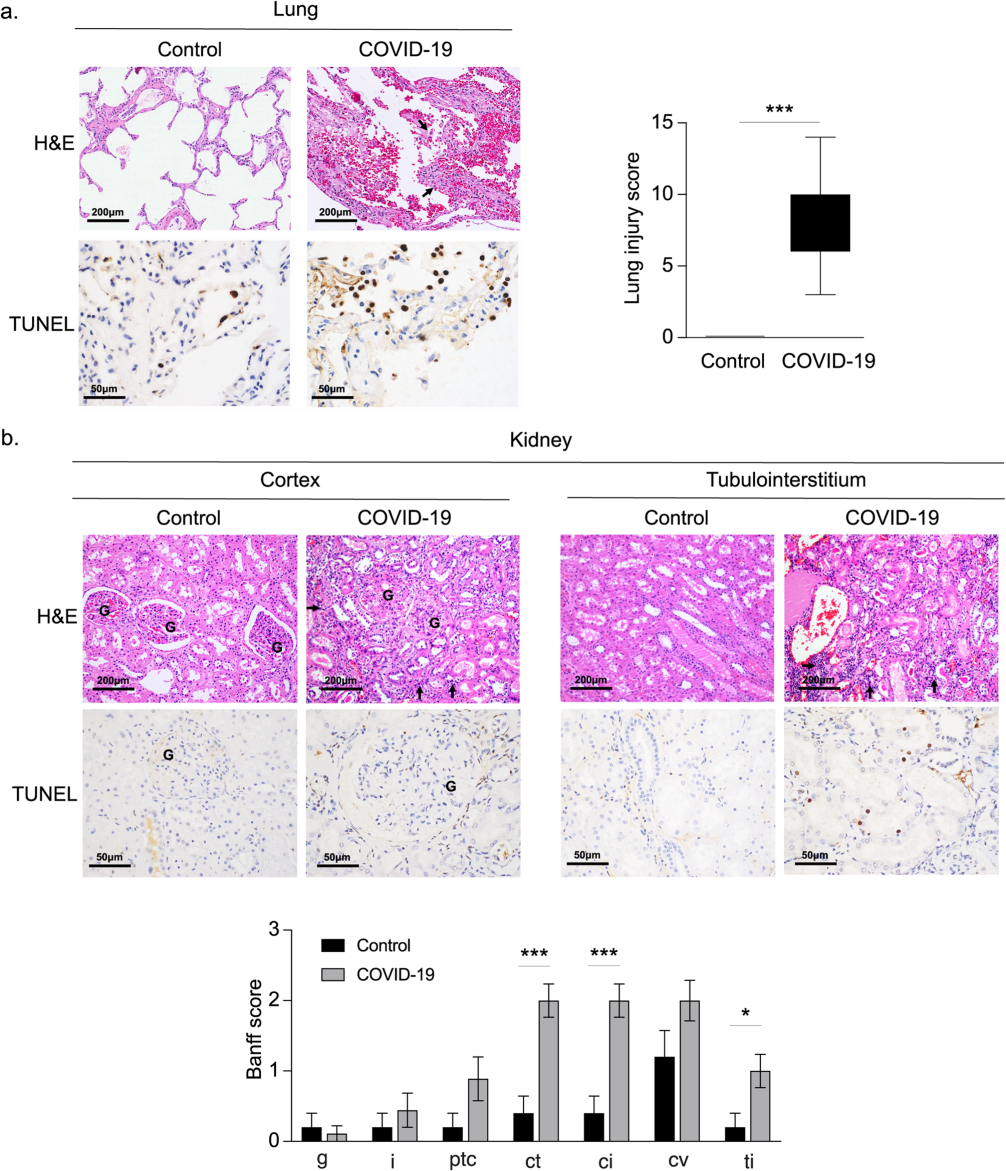
Histopathology findings of lung and kidney in COVID19 patients and controls. Representative H&E and TUNEL staining in (**a**) lung and (**b**) kidney tisuses from patients with COVI19 (COVID-19, n = 9) and controls (Control, n = 5). Black arrows indicate mononuclear inflammatory cells. Lung injury was assessed on a scale of 0–2 for each of the following criteria: (i) alveolar polymorphonuclear neutrophils, (ii) chronic alveolar inflammation/macrophages, (iii) acute alveolar wall Inflammation, (iv) chronic alveolar wall inflammation, (v) hyaline membranes, (vi) Type 2 hyperplasia only, (vii) Type 2 hyperplasia with fibroblasts, and (viii) organizing pneumonia and squamous metaplasia. The final injury score was derived from the following calculation: Score = I + ii + iii + iv + v + vi + vii + viii. G indicates glomerulus. Banff Score: g, glomerulitis; i, interstitial inflammation; ptc, peritubular capillaritis; ct, tubular atrophy; ci, interstitial fibrosis; cv, vascular fibrous intimal thickening; ti, total inflammation. *p < 0.05, **p < 0.01.

We have demonstrated that higher levels of inflammatory cytokines and chemokines during COVID-19 infection are associated with disease severity and death. Since organ-specific injury such as lung and kidney^17^ may be a contributing factor, we examined histopathological features of lung and kidney specimen from COVID-19 patients in the cohort who died during the index hospitalization. Baseline demographics are shown in Supplementary Table 9. Histopathological examination by H&E staining of lung showed that COVID-19 patients exhibit diffuse alveolar damage with significant mononuclear inflammatory cell infiltration (Fig. 6a). Additionally, the lungs of COVID-19 patients demonstrate greater epithelial cell injury in comparison to controls, indicated by an increase in the number of terminal deoxynucleotidyl transferase dUTP nick end labeling (TUNEL) positive cells. In COVID-19 kidneys, progressive glomerular sclerosis, tubulointerstitial lymphocyte infiltration and moderate to severe cortical and medullary atrophy were observed (Fig. 6b). As with lung, TUNEL positive cells were increased in periglomerular and tubular epithelial cells of COVID-19 patients, suggesting that COVID-19 infection may cause glomerular and renal tubular injury (Fig. 6b). The pathologic features of cortex and tubulointerstitium in COVID-19 kidney and controls in each Banff scoring is shown in Fig. 6b. Total inflammation (*ti*) was elevated in COVID-19 patients compared to controls. Chronic changes such as tubular atrophy (*ct*) and interstitial fibrosis (*ci*) were also significantly increased in COVID-19 cases (Fig. 6b).

## Discussion

This study highlights important cytokine expression differences between COVID-19 patients and similarly presenting, contemporaneously admitted control patients. Within the COVID-19 cohort, we found numerous correlations with the baseline burden of organ dysfunction as well as with the subsequent development of critical clinical outcomes of ARDS, AKI, and death. We revealed patterns of dysregulated cytokines associated with the presence of COVID-19 disease, its severity, and subsequent lung and kidney injury^6, 18^**.** However, we did not find substantial variation in our cytokine panel across the first three days in our COVID-19 group.

In our analysis of admission cytokines and baseline organ injury, several Th1 and Th2 associated cytokines were correlated to SOFA score in COVID-19 patients as shown in Fig. 4. This suggests potential imbalances in Th1 and Th2 driven immunity, which could lead to altered neutrophil recruitment, monocyte and epithelial activation. This in turn supports the increased mononuclear inflammatory infiltrates in the histopathology findings present in fatal COVID-19 cases^19^. These findings, in combination with the apparent stability in the cytokine panel over time, suggest that the proposed pathophysiological response is persistent. We associated cytokine signatures at admission with histopathological findings from the same patients that ultimately succumbed to COVID-19 during their index admission. Our autopsy findings of COVID-19 patients demonstrate mononuclear inflammatory cell infiltration such as macrophages and lymphocytes in both lung and kidney tissues. This is consistent with the findings of increased macrophage and lymphocyte derived cytokines in plasma samples of COVID-19 patients compared to controls.

Given the accumulation of evidence of the systemic effects of COVID-19, we analyzed a broad cytokine panel’s relationship with the subsequent development of ARDS, AKI, and death. ARDS and AKI in COVID-19 are associated with a high morbidity even without mortality^6, 7, 9, 20^ and the role of cytokines in the development of these complications remains poorly understood. Our data shows that inflammatory cytokine signatures are associated with systemic disease severity beyond pneumonia. Specifically, the development of both ARDS and AKI in COVID-19 patients is associated with IL-1RA, fractalkine, M-CSF, G-CSF, IL-6, and TNF-α, and supports the potential deleterious effect of the “cytokine storm” on disease progression^15, 21^. IL-1RA, which is mostly produced by epithelial cells, can also be made by multiple types of immune cells and by binding to the IL-1R can act as a natural inhibitor of IL-1ß. Given the association with both ARDS and AKI, it is plausible that IL-1RA is potentially associated with changes in pulmonary function, lung damage, and increased kidney injury. Interestingly, IL-1RA plays an important role in lipid metabolism, fever generation, neutrophil chemotaxis, positive regulation of IL-6 production, and the acute-phase response of infection. As epithelial cells and macrophages are the main producers of IL-6 in the lung, elevated IL-1RA expression may further augment IL-6 production by these cells; thereby, contributing to a deleterious positive feedback loop within COVID-19 patients that can directly impact both the lung and kidney. Further, as demonstrated by our results, heightened TNF-α production was indicative of severe disease progression in COVID-19 patients. As there is an inverse relationship between TNF-α expression and T cell recruitment, similar to IL-1RA and IL-6, elevated TNF-α expression may further contribute to inflammatory progression of COVID-19 patients to develop ARDS or AKI. However, increased IL-18 on day 1, a marker for inflammasome activation, was inversely associated with mortality. Appropriate inflammasome activation may play a critical role in the host defense during SARS-CoV-2 infection^22^. Pyropstosis, an inflammasome and casapase-1 mediated programmed cell death, has been shown to play an important role in viral diseases^23^. Inflammasome recognition of viral molecules can lead to the activation of pyroptosis by promoting caspase-1 activation and IL-1β and IL-18^23^. Additionally, Inflammasome impairment have been shown to decrease survival in elderly mice during Influenza infection^24^.

Our study also demonstrated levels of chemokines, such as G-CSF and M-CSF, correlated with the development of ARDS and AKI in COVID-19 patients, suggesting a role of leukocyte maturation and activation in disease progression. M-CSF is a primary chemokine associated with the growth, proliferation, and differentiation of hematopoetic cells, including monoblasts, pro-monocytes, monocytes, macrophages, and osteoclasts. M-CSF is secreted by monocytes, fibroblasts, stromal cells, and endothelial cells^25^. As demonstrated in our current findings, higher levels of M-CSF were not only associated with pneumonia severity, but also highlighted the potential for monocyte/macrophage driven development of either ARDS or AKI. GCSF is produced by macrophages and the endothelium and is essential for the proliferation and maturation of neutrophils, eosinophils, and basophils. In response to elevated G-CSF, proliferation and differentiation of precursor cells into mature granulocytes occurs^26^. Mature granulocytes, play an important role in chemotaxis, phagocytosis, as well as the release of lysosomal enzymes at sites of infection. Increased G-CSF can occur in patients with neutropenia as a feedback mechanism to increase neutrophil migration to the site of infection. While these cells are essential for the host antiviral response, an overzealous or heightened response can lead to increased cellular death and loss of homeostasis. Based on our current findings, we hypothesize that the cellular dysfunction and death in the lung in severe COVID-19 modifies the initiation and regulation of an ‘effective’ innate immune response. Importantly, as these cytokine signatures are present in plasma, it is plausible that this dysregulated, overly zealous immune response can result in systemic organ dysfunction.

We found that MCP-1, MCP-3, IP-10, and IL-8, which associated with ARDS, were not associated with AKI and mortality, suggesting a differential role in monocyte migration and macrophage activation in disease development. MCP-1 is a powerful monocyte chemotactic factor that is constitutively produced by oxidative stress, cytokines, or growth factors and can be expressed by endothelial cells, fibroblasts, epithelial cells, monocytes, and macrophages. Similar to M-CSF, MCP-1 plays an important role in the antiviral response and regulates the migration and infiltration of monocytes and NK cells^27^. MCP-3 is produced by macrophages and attracts monocytes to the site of infection^28^. MCP-3 regulates macrophage function through its binding to chemokine receptors CCR1, CCR2, and CCR3. IL-8 can act as a chemoattractant to recruit neutrophils and other immune cells to the site of infection. IL-8 is secreted by macrophages, but can also be released by epithelial cells, airway smooth muscle cells, and endothelial cells. Of note, IL-8 is involved in multiple cellular processes, such as tissue proliferation, tissue remodeling, and angiogenesis^29^. IP-10 is secreted by neutrophils, endothelial cells, fibroblasts, dendritic cells, and hepatocytes. IP-10 binds to CXCR3 to regulate immune system responses by activating and recruiting leukocytes, including T cells, monocytes, and NK cells. Recruitment of leukocytes to inflamed tissues can perpetuate inflammation, and thereby, increased IP-10 can contribute to extensive tissue damage^30^. In summary, as detailed by our data, heightened expression of MCP-1, MCP-3, IL-8, and/or IP-10 demonstrates how an overzealous monocyte/macrophage driven immune response can contribute to ARDS development in COVID-19.

Although prior studies have noted the association of increased pro-inflammatory cytokines in COVID-19 pneumonia severity^10–13, 31^, we found distinct cytokine signatures for eventual ARDS, AKI and mortality. In contrast to mortality, we observed that ARDS and AKI development were associated with macrophage migration, and immune cell and epithelial activation, which suggests an exclusive Th1 driven immunity by TNF-β and IFN-α2.

We saw less correlations with between novel cytokines, clinical labs, and severity of illness in our control patients. This result supports a more homogeneous but graded dysregulated immune response in COVID-19. The increased proportion of patients with cancer and immunosuppression in the control population does yield potential to bias our results, however, cancer typically elicits an inflammatory response. Moreover, We did not explore cytokines within urine samples in this study. Additionally, it is important to mention that standard treatment for COVID-19 at the time of our sample collection did not include routine use of dexamethasone or remdesivir which may alter the relationship between cytokines and eventual outcomes. Moreover, the surge conditions in New York City may have affected the clinical practice pattern during the study period. However, our in-hospital mortality was similar to other reports from around the United States and the world^3, 7^.

Overall, our findings support the role of dysregulate broad cytokine response in the pathogenesis of severe COVID-19. Specifically, our study provides an extensive analysis on how cytokine signatures differentially associate with baseline organ failure, clinical labs, eventual disease severity, and organ specific complications of ARDS and AKI. These findings highlight potential leukocyte and monocyte specific immunologic signatures that suggest differential pathophysiology leading to AKI, ARDS, and mortality. Cytokine signatures could be used for the prognostication of specific types of organ dysfunction. They also suggest alternative therapies for patients with COVID-19.

## Methods

### Human subjects and design

Our cohort study included adults 18 years of age or older with confirmed COVID-19 and a population of SARS-CoV-2 negative controls who were admitted to the general wards between March 3, 2020 (date of the first case) and April 1, 2020 at an 862-bed quaternary referral center in New York City. All patients presented to the emergency department for an acute complaint and were subsequently admitted for inpatient care. COVID-19 cases had presenting symptoms, fever, cough, and dyspnea consistent with COVID-19 and were confirmed through reverse transcriptase polymerase chain reaction (rt-PCR) assay for SARS-CoV-2, performed on nasopharyngeal swab specimens. Control patients were made up of contemporaneous admissions to the hospital who had a negative rt-PCR for SARS-CoV-2 and were not considered to have an illness consistent with COVID-19. Pediatric and pregnant patients were excluded from the study. Additionally, autopsy specimen, if available, from patients in the cohort were included. The study was approved by the institutional review board of Weill Cornell Medicine (20-05022072, 19-10020914, 20-04021880, 20-04021796, and 20-03021681) with a waiver of informed consent for serum specimens and autopsy consent was obtained by next of kin which includes informed consent for study participation. All methods were performed in accordance with the relevant guidelines and regulations.

### Clinical evaluation

Baseline demographics, clinical characteristics, comorbid conditions, vital signs, laboratory values, and radiographic findings on presentation were manually abstracted from the electronic health record by trained research personnel with the use of a quality-controlled protocol and structured abstraction tool^4^. Laboratory and radiographic testing were performed according to clinical needs and analyzed/interpreted on site. The Sequential Organ Failure Assessment (SOFA) score, a severity of illness score that sums six separate organ dysfunction subscores, was used to characterize baseline severity of illness. For the central nervous system, kidney, liver, and coagulation organ dysfunction subscores, traditional SOFA methodology^32^ was used. When the respiratory SOFA subscore was not available due to a lack of partial pressure of oxygen (PaO_2_), we used a commonly accepted imputation technique to impute PaO_2_ from an oxygen saturation (SpO_2_) level^33^. The cardiovascular SOFA subscore was updated with additional vasopressors according to a norepinephrine equivalency table^34^. Missing data for each subscore was treated as normal. We additionally classified COVID-19 patients as moderate or severe based on the World Health Organization (WHO) interim guidelines system^35^, by in-hospital maximal oxygen and organ failure support. Acute kidney injury (AKI) was defined by the Kidney Disease Improving Global Outcomes (KDIGO) criteria and staging^36^. ARDS was defined according to the Berlin definition^37^ as the need for mechanical ventilation, bilateral infiltrates in the chest x-ray, and clinical diagnosis of ARDS by the treating attending physician. Thromboembolic events included any deep vein thrombosis or pulmonary embolism confirmed radiographic imaging. Respiratory co-infections included any other viral, bacterial, or fungal pathogen isolated on any respiratory sample (e.g., nasopharyngeal swab, sputum sample, bronchoalveolar lavage).

### Measurement of biomarkers

Serum specimens of COVID-19 cases and control patients were obtained from the clinical laboratory for each patient on day one, two, and three after admission to the general ward floor. Please refer to Supplemental Methods for detailed serum isolation protocols. Briefly, serum was collected after centrifugation of whole blood at 1500*g* for seven minutes at room temperature. The serum was then aliquoted and stored at − 80 °C. Serum samples were shipped to the Advanced Diagnostics Laboratories at National Jewish Health (Denver, Colorado) on dry ice, and concentrations of cytokines and chemokines were measured using the Human Cytokine/Chemokine 8-plex Assay (HCYTOMAG-60K, Millipore) and the Cytokine/Chemokine/Growth Factor 48-plex Panel (HCYTA-60K-PX48, Millipore) on a Luminex MAGPIX instrument system, following the manufacturer’s protocol.

### Statistics

Baseline demographics, comorbid conditions, severity of illness, and clinical outcomes between the COVID-19 and control patients were compared using Kruskal–Wallis, Chi-square, and Fisher’s exact tests as appropriate. The same patient level differences were analyzed between a COVID-19 cohort with repeat (days one, two, and three of hospital admission) Luminex samples and the larger COVID-19 cohort. Cytokines with greater than > 80% of values outside of the detectable assay range for the COVID-19 cohort were removed from the analysis. All remaining cytokines were assessed for skewness and ultimately analyzed on the log_2_ scale, with cytokines outside of the detectable range analyzed conservatively at the limit of detection. With the exception of baseline demographic comparisons, p-values accompanying each analysis were adjusted for multiple comparisons using the Benjamini Hochberg False Discovery Rate (FDR) correction. A significance threshold of < 0.05 after FDR adjustment of the p-value (denoted as the q-value in results) was used. The 95% Confidence Intervals (CI) of estimates were computed as appropriate.

#### Day one samples

Differences between cytokine levels on day one of hospital admission in COVID-19 and control patients were first estimated using linear regression models with robust standard errors. Next, a correlation matrix of all day one cytokines was computed within COVID-19 and control patients. Each day 1 cytokine level was additionally correlated with the day one of hospital admission SOFA score within COVID-19 and control patients. Finally, clinical laboratory tests (platelet count, procalcitonin, lactate dehydrogenase, ferritin, d-dimer, C-reactive protein, the international normalization ratio, and serum creatinine) available within the first 72 h of admission were correlated with the day one of hospital cytokine levels. In the event of multiple laboratory tests, the first was used, and in the case of missing laboratory tests, a complete case correlation was calculated. The non-parametric Spearman’s rank formula was used to estimate all correlation coefficients.

Lastly, time-to-event analyses for the clinical outcomes of interest were performed for each cytokine. Time of event for the ARDS and mortality outcomes were determined by the length of time to intubation or death relative to hospital admission, and patients who did not experience the event were censored at discharged. For the outcome of AKI, patients who died before meeting the criteria for AKI were additionally censored at the time of death to yield a cause-specific hazard ratio. All outcome associations were estimated using Cox Proportional Hazard (PH) models with robust standard errors.

#### Day one, two, and three samples

A subset of COVID-19 patients with day one, two, and three Luminex 48-plex samples were tested for differences across time with the non-parametric Freidman’s test for repeated measures. A larger subset of COVID-19 patients with Luminex 8-plex samples from day one, two, and three were analyzed in the same way.

#### Autopsy specimen

Autopsies of COVID-19 patients were done in accordance with CDC guidelines following consent obtained by the next of kin. The lungs were inflated with formalin prior to sectioning and all tissues were fixed in 10% buffered formaldehyde for 24–48 h prior to routine processing. Paraffin sections were stained with hematoxylin and eosin for histologic interpretation and immunohistochemical staining was done for TdT-mediated dUTP Nick-End Labeling (TUNEL) evaluation. Stained lung sections were analyzed by Olympus BX53M microscope and four regions of interest evaluated for each case. Eight COVID-19 cases and three normal controls were of sufficient quality to score. Images were imported into ImageJ and color deconvolution was performed using the appropriate plug-in for H-DAB. Positive cells for TUNEL were counted on one channel while total cells on the other channel. Autopsy lung and kidney tissue results were averaged per case. All autopsy data were analyzed using T-Tests in SPSS v 25, while the box and whisker plot were generated in Microsoft Excel. All other statistical analyses unrelated to the autopsy data were performed in R version 3.6.3 and figures were generated using the package ggplot2.

## Supplementary Information

Supplementary Information.

## Data Availability

Deidentified individual participant data that underlie the results reported in this manuscript will be available to the scientific community on request. Applicants interested must provide a proposal form which entails the scientific aim of the usage of the data provided and the institutional review board approval of the research proposal. All proposals should be directed to the corresponding author. Data will be available indefinitely after publication of the manuscript.
