# Supplementary material for: Cytokine signatures of end organ injury in COVID-19
Source: Sci Rep. 2021 Jun 15;11:12606. doi: 10.1038/s41598-021-91859-z (PMC8206105; doi:10.1038/s41598-021-91859-z)
Supplement: Supplementary file 1 — Supplementary Information. [file 41598_2021_91859_MOESM1_ESM.pdf]

Cytokine Signatures of End Organ Injury in COVID-19

Luis G. Gómez-Escobar[1], Katherine L. Hoffman[2], Justin J. Choi[3], Alain Borczuk[4], Steven Salvatore[4], Sergio L. Alvarez-Mulett[1], Manuel D. Galvan[5], Zhen Zhao[4], Sabrina E. Racine-Brzostek[4], He S. Yang[4], Heather Winona Stout-Delgado[1], Mary E Choi[6], Augustine MK Choi[1], Soo Jung Cho[1*], Edward J. Schenck[1*]

[1] Division of Pulmonary and Critical Care Medicine, Department of Medicine, Weill Cornell Medicine, New York, New York.

[2] Division of Biostatistics, Department of Population Health Sciences, Weill Cornell Medicine, New York, New York.

[3] Department of Medicine, Weill Cornell Medicine, New York, New York.

[4] Department of Pathology and Laboratory Medicine, Weill Cornell Medicine, New York, New York.

[5] Advanced Diagnostics Laboratories, National Jewish Health, Denver, Colorado.

[6] Division of Nephrology and Hypertension, Department of Medicine, Weill Cornell Medicine, New York, New York.

*** Corresponding Authors. These authors jointly supervised this work.**

Correspondence to:

Edward J. Schenck

Division of Pulmonary and Critical Care Medicine

1300 York Avenue

New York, NY 10065

Phone : 212-746-2262

Email : ejs9005@med.cornell.edu

Supplementary Materials.

Serum Isolation Protocol

Remnant serum specimen from COVID 19 RT-PCR positive patients between March 03, 2020 and April 01, 2020 were collected and stored at -80°C until further analysis. Any patients with remnant serum from at least one Serum Separator Tube (SST) during the time period of 72 hours after admission were included in the study. Standard practices for serum collection and storage at NewYork Presbyterian/Weill Cornell Medical College include collecting venous blood into an SST tube and serum is physically separated from cells (centrifuged at 1500 g for 7 minutes) as soon as possible with a maximum limit of 2 hours from the time of collection. The specimen are typically stored at 4C for 1-5 days before coded/deidentified, transferred to a new coded tube and frozen at -80C. When serum specimen were needed for additional testing, the tube was retrieved and thawed at 4C, at which point the necessary aliquots were taken for testing.

Supplementary Table 1. Admission Diagnosis COVID-19 negative patients.

Characteristic	COVID-19 Negative (N=51)
Sepsis-suspected	2
Pneumonia	10
Heart failure	3
Acute Respiratory Failure	3
Altered Mental Status	1
Drug/alcohol overdose	1
Asthma exacerbation	1
Chest pain	
Cardiovascular	
Acute myocardial infarction	2
Unstable angina	2
Pericarditis/cardiac tamponade	1
Pleuritic	1
Acute kidney injury	3
Other infection	
Blood	3
Genito/urinary tract	1
Respiratory tract other than pneumonia	4
Gastrointestinal tract	1
Trauma	1

Other	11

Supplementary Table 2. Demographics and baseline characteristics COVID-19 patients with repetitive measures (Day 1, 2 and 3). P-values are computed from Kruskal-Wallis, Fisher's Exact, or Chi-square tests, as appropriate.

Characteristic	COVID-19 Positive (N=90)	COVID-19 Positive with repeat samples (N=15)	p.value
**Demographics**			
Age (median, IQR)	66 (57, 77)	63 (59, 75)	0.7
Sex (n, %)			0.3
Female	33 (37%)	3 (20%)	
Male	57 (63%)	12 (80%)	
Race (n, %)			>0.9
Asian	6 (7.5%)	1 (7.7%)	
Black	12 (15%)	2 (15%)	
Other	18 (22%)	2 (15%)	
White	44 (55%)	8 (62%)	
BMI (median, IQR)	28 (23, 31)	28 (24, 31)	>0.9
Smoking status (n, %)			0.4
Former Smoker	30 (33%)	3 (20%)	
No	60 (67%)	12 (80%)	
**Comorbidities** (n, %)			
CAD	13 (14%)	1 (6.7%)	0.7
DM	28 (31%)	4 (27%)	>0.9
HTN	51 (57%)	7 (47%)	0.7
CVA	9 (10%)	2 (13%)	0.7
CKD/ESRD	9 (10%)	1 (6.7%)	>0.9
Cirrhosis	1 (1.1%)	1 (6.7%)	0.3
COPD	6 (6.7%)	0 (0%)	0.6
Asthma	10 (11%)	1 (6.7%)	>0.9
Active cancer	11 (12%)	2 (13%)	>0.9
Immunosuppressed state	4 (4.4%)	2 (13%)	0.2
**Home Medications** (n, %)			
Immunosupression medications (past 30 days)	15 (17%)	6 (40%)	0.073
ACE/ARBs	25 (28%)	5 (33%)	0.8
Statins	33 (37%)	6 (40%)	>0.9
NSAIDs	24 (27%)	2 (13%)	0.3
PPIs	21 (23%)	3 (20%)	>0.9

Supplementary Table 3. Median and interquartile range of expression of inflammatory cytokines of COVID-19 patients with repetitive measures (Day 1, 2 and 3). Q-value represents the p-value computed from Freidman's test for differences across days after adjustment for multiple comparisons. **Relative to Supplemental Figure 1.**

Cytokine	Day 1	Day 2	Day 3	q-value
**TNF-β**	9.5 (8.0, 13.3)	8.0 (8.0, 11.8)	8.0 (8.0, 14.0)	0.47
**TNF-α**	99 (65, 119)	97 (76, 109)	79 (66, 96)	0.72
**TGF-α**	14 (10, 23)	10 (7, 19)	11 (9, 18)	0.96
**sCD40L**	1022 (609, 2220)	1201 (451, 1550)	1397 (803, 2437)	0.96
**PDGF-AB/PDGF-BB**	30028 (27038, 33606)	31935 (23271, 33519)	31975 (21838, 32921)	0.96
**PDGF-AA**	4168 (3060, 5631)	4383 (3142, 4760)	4216 (2337, 5256)	0.95
**CCL4/MIP-1β**	49 (34, 56)	49 (41, 67)	48 (40, 66)	0.95
**CCL3/MIP-1α**	19 (16, 29)	18 (16, 31)	16 (16, 24)	0.96
**CXCL9/MIG**	3855 (2420, 5506)	4404 (2603, 6940)	4476 (2781, 6304)	0.85
**CCL22/MDC**	472 (348, 625)	372 (277, 576)	342 (269, 537)	0.47
**CCL7/MCP3**	43 (40, 79)	62 (40, 79)	54 (44, 99)	0.47
**CCL2/MCP1**	1272 (902, 2103)	1362 (847, 2556)	1582 (1055, 2552)	0.2
**CSF-1/M-CSF**	518 (460, 582)	547 (342, 690)	515 (411, 650)	0.95
**CXCL10/IP-10**	3849 (2353, 9403)	3769 (2340, 8585)	2793 (1032, 7075)	0.85
**IFN-γ**	31 (15, 45)	33 (17, 46)	21 (18, 34)	0.96
**IFN-α2**	40 (40, 42)	40 (40, 70)	40 (40, 40)	0.95
**IL-9**	15 (9, 20)	14 (10, 19)	15 (12, 19)	0.95
**IL-8**	32 (23, 56)	37 (24, 48)	32 (29, 55)	0.96
**IL-7**	11.8 (5.8, 16.1)	11.7 (7.5, 15.4)	10.0 (3.9, 14.5)	0.95
**IL-6**	130 (54, 188)	81 (56, 144)	136 (83, 194)	0.85
**IL-5**	3.9 (3.2, 4.7)	4.0 (3.4, 7.1)	5.6 (3.8, 11.7)	0.72
**IL-4**	3.20 (3.20, 3.20)	3.20 (3.20, 3.20)	3.20 (3.20, 3.20)	0.85
**IL-27**	4614 (2334, 5718)	4517 (2487, 7012)	4284 (2076, 9188)	0.96
**IL-1RA**	51 (27, 80)	41 (23, 397)	31 (21, 64)	0.95
**IL-1β**	8 (8, 17)	11 (8, 16)	8 (8, 13)	0.95
**IL-18**	64 (47, 132)	64 (46, 140)	101 (57, 149)	0.47
**IL-17E/IL-25**	459 (359, 563)	458 (360, 556)	363 (278, 547)	0.47
**IL-17A**	6.4 (6.4, 10.0)	6.4 (6.4, 8.4)	6.4 (6.4, 8.0)	0.96
**IL-15**	30 (19, 36)	28 (20, 37)	26 (21, 39)	1
**IL-13**	32.0 (32.0, 35.1)	32.0 (32.0, 32.0)	32.0 (32.0, 32.0)	0.95
**IL-12 (p40)**	53 (32, 81)	58 (37, 83)	52 (36, 78)	0.96
**IL-10**	47 (32, 68)	58 (32, 91)	40 (28, 77)	0.95
**CXCL1/GROα**	55 (44, 64)	46 (25, 65)	40 (30, 58)	0.95

**G-CSF**	78 (53, 139)	77 (32, 110)	34 (24, 84)	0.47
**Fractalkine**	160 (160, 185)	160 (160, 207)	160 (160, 169)	0.95
**FLT-3L**	30 (22, 37)	29 (23, 34)	28 (23, 33)	0.96
**FGF-2**	198 (166, 265)	194 (170, 258)	223 (163, 254)	0.96
**Eotaxin**	146 (73, 168)	127 (84, 159)	105 (70, 168)	0.96
**EGF**	59 (33, 99)	39 (27, 104)	42 (26, 95)	0.95

Supplementary Table 4. 8-Plex Cytokine differences in COVID-19 patients compared to controls.
8-Plex Cytokine differences in COVID-19 patients compared to controls.

Cytokine	Log FC (95% CI)	q-value
**IL-10**	1.37 (0.59, 2.14)	0.003
**IL-6**	1.04 (0.17, 1.9)	0.047
**IFN-γ**	0.72 (0.07, 1.38)	0.049
**TNF-α**	0.49 (-0.05, 1.02)	0.091
**IL-4**	-0.34 (-1.42, 0.74)	0.539

a. COVID-19 patients cytokine expression by repeat measures (day 1, 2, and 3).

Cytokine	Day 1	Day 2	Day 3	q-value
**TNF-α**	15 (9, 29)	16 (10, 37)	18 (8, 39)	0.48
**IL-6**	26 (11, 70)	25 (13, 62)	25 (9, 73)	0.45
**IL-4**	3 (3, 11)	3 (3, 8)	3 (3, 6)	0.36
**IL-10**	31 (12, 53)	35 (15, 56)	29 (12, 50)	0.48
**IFN-γ**	8 (3, 24)	8 (3, 24)	4 (3, 20)	0.31

Supplementary Table 5. Expression of inflammatory cytokines in COVID-19 patients compared to controls on day 1 of hospital admission. Estimates and 95% Confidence Intervals (CI) are computed from linear regression models with robust standard errors. Q-value represents the p-value after adjustment for multiple comparisons. Relative to **Figure 1 and Supplemental Figure 1.**

Cytokine	Log FC (95% CI)	q-value
**CXCL10/IP-10**	3.52 (2.85, 4.19)	**<.001**
**TNF-α**	0.5 (0.28, 0.71)	**<.001**
**IFN-α2**	0.36 (0.2, 0.52)	**<.001**
**IL-1RA**	1.24 (0.66, 1.82)	**<.001**
**IFN-γ**	0.99 (0.48, 1.51)	**0.001**
**CSF-1/M-CSF**	0.48 (0.2, 0.75)	**0.005**
**CCL2/MCP1**	0.63 (0.26, 1)	**0.005**
**CCL7/MCP3**	0.15 (0.06, 0.24)	**0.008**
**IL-7**	0.49 (0.17, 0.81)	**0.011**
**IL-15**	0.27 (0.07, 0.46)	**0.029**
**IL-12 (p40)**	0.41 (0.1, 0.72)	**0.037**
**IL-6**	0.98 (0.19, 1.76)	**0.048**

**CCL4/MIP-1β**	-0.48 (-0.87, -0.09)	**0.048**
**IL-10**	0.58 (0.07, 1.1)	0.073
**FLT-3L**	0.41 (0.01, 0.82)	0.117
**Fractalkine**	0.1 (0, 0.2)	0.139
**CCL22/MDC**	-0.3 (-0.62, 0.01)	0.139
**TNF-β**	0.14 (-0.02, 0.29)	0.168
**PDGF-AA**	0.37 (-0.07, 0.81)	0.205
**IL-17A**	0.18 (-0.05, 0.4)	0.221
**IL-18**	0.41 (-0.1, 0.92)	0.221
**sCD40L**	-0.47 (-1.08, 0.14)	0.233
**IL-5**	-0.28 (-0.65, 0.09)	0.233
**CXCL1/GROα**	0.32 (-0.26, 0.89)	0.461
**IL-17E/IL-25**	0.14 (-0.13, 0.42)	0.492
**IL-4**	0.08 (-0.09, 0.25)	0.567
**FGF-2**	0.07 (-0.13, 0.27)	0.701
**PDGF-AB/PDGF-BB**	0.12 (-0.23, 0.48)	0.701
**IL-8**	0.18 (-0.41, 0.77)	0.733
**IL-1β**	0.09 (-0.22, 0.4)	0.737
**IL-27**	-0.11 (-0.62, 0.41)	0.865
**CXCL9/MIG**	-0.08 (-0.62, 0.46)	0.909
**CCL3/MIP-1α**	-0.06 (-0.47, 0.35)	0.909
**Eotaxin**	0.03 (-0.31, 0.37)	0.96
**TGF-α**	-0.04 (-0.45, 0.37)	0.96
**G-CSF**	-0.04 (-0.63, 0.56)	0.969
**IL-9**	-0.01 (-0.37, 0.34)	0.969
**IL-13**	0 (-0.11, 0.12)	0.969
**EGF**	0 (-0.42, 0.41)	0.995

Supplementary Table 6. Expression of inflammatory cytokines in COVID-19 mild and severe cases on day 1 of hospital admission. Estimates and 95% Confidence Intervals (CI) are computed from linear regression models with robust standard errors. Q-value represents the p-value after adjustment for multiple comparisons. Relative to **Figure 2.**

Cytokine	Log FC (95% CI)	q-value
**IL-6**	1.54 (0.85, 2.24)	**<.001**
**CXCL10/IP-10**	1.73 (0.98, 2.49)	**<.001**
**TNF-α**	0.65 (0.35, 0.96)	**<.001**
**IL-1RA**	1.45 (0.67, 2.22)	**0.003**
**IL-8**	0.89 (0.38, 1.41)	**0.004**
**CSF-1/M-CSF**	0.55 (0.24, 0.87)	**0.004**
**G-CSF**	0.98 (0.34, 1.62)	**0.015**
**CCL2/MCP1**	0.58 (0.2, 0.97)	**0.015**
**CCL7/MCP3**	0.26 (0.08, 0.45)	**0.02**

**PDGF-AA**	-0.55 (-0.95, -0.15)	**0.028**
**PDGF-AB/PDGF-BB**	-0.4 (-0.71, -0.08)	**0.045**
**IL-10**	0.68 (0.11, 1.25)	**0.059**
**IL-15**	0.38 (0.06, 0.71)	**0.059**
**IL-27**	0.52 (-0.03, 1.08)	0.181
**FLT-3L**	0.32 (-0.03, 0.66)	0.187
**Fractalkine**	0.15 (-0.02, 0.32)	0.187
**FGF-2**	0.25 (-0.08, 0.58)	0.301
**IL-18**	0.41 (-0.13, 0.95)	0.301
**Eotaxin**	0.27 (-0.11, 0.64)	0.311
**IL-9**	0.32 (-0.13, 0.76)	0.311
**CCL22/MDC**	-0.24 (-0.6, 0.12)	0.344
**sCD40L**	0.46 (-0.28, 1.21)	0.347
**EGF**	-0.3 (-0.76, 0.17)	0.347
**CCL4/MIP-1β**	0.2 (-0.12, 0.52)	0.347
**TGF-α**	0.4 (-0.21, 1)	0.347
**IL-13**	0.11 (-0.08, 0.29)	0.402
**CXCL1/GROα**	0.34 (-0.3, 0.97)	0.422
**TNF-β**	0.14 (-0.13, 0.42)	0.422
**IL-17E/IL-25**	0.19 (-0.22, 0.6)	0.488
**IL-5**	0.16 (-0.21, 0.52)	0.526
**IL-1β**	0.17 (-0.29, 0.63)	0.591
**IL-4**	-0.09 (-0.35, 0.17)	0.597
**IL-12 (p40)**	0.16 (-0.31, 0.63)	0.603
**CXCL9/MIG**	0.14 (-0.34, 0.62)	0.657
**IFN-α2**	0.05 (-0.27, 0.38)	0.842
**IL-7**	-0.06 (-0.5, 0.38)	0.842
**IL-17A**	0.06 (-0.36, 0.48)	0.842
**IFN-γ**	0.03 (-0.65, 0.7)	0.962
**CCL3/MIP-1α**	0.01 (-0.44, 0.46)	0.964

Supplementary Table 7. Significant clinical laboratory and cytokine correlations in COVID-19 patients and controls. Correlation coefficients are estimated using Spearman's correlation formula. All significant ($p < 0.05$) correlation pairs prior to adjustment for multiple comparisons are shown. Q-value represents the p-value after adjustment for multiple comparisons. Relative to **Figure 3**.

a. Among controls

Cytokine	Lab	N	Spearman's Correlation	p-value	q-value
**PDGF-AB/PDGF-BB**	Platelet	51	0.78	<.001	**<.001**
**PDGF-AA**	Platelet	51	0.71	<.001	**<.001**
**VEGF-A**	Platelet	51	0.58	<.001	**<.001**
**CSF-1/M-CSF**	Creatinine	51	0.46	<.001	**0.05**

**CCL2/MCP1**	Creatinine	51	0.44	0.001	**0.08**
**IL-7**	Platelet	51	0.41	0.003	0.13
**IL-13**	Ferritin	7	-0.93	0.003	0.13
**PDGF-AB/PDGF-BB**	INR	32	-0.5	0.004	0.14
**PDGF-AA**	INR	32	-0.5	0.004	0.14
**IL-1β**	Ferritin	7	-0.91	0.005	0.14
**IL-17E/IL-25**	Platelet	51	0.38	0.005	0.14
**IL-10**	Platelet	51	-0.38	0.006	0.14
**TGF-α**	Ferritin	7	-0.9	0.006	0.14
**CCL22/MDC**	Platelet	51	0.38	0.007	0.15
**IL-27**	Procalcitonin	15	0.67	0.007	0.15
**TNF-α**	Platelet	51	-0.37	0.008	0.15
**CXCL9/MIG**	Platelet	51	-0.37	0.008	0.15
**IL-1β**	Procalcitonin	15	-0.64	0.009	0.17
**CXCL10/IP-10**	Creatinine	51	0.36	0.01	0.17
**EGF**	Platelet	51	0.35	0.01	0.19
**CSF-1/M-CSF**	Procalcitonin	15	0.63	0.01	0.19
**PDGF-AB/PDGF-BB**	C-Reactive Protein	7	-0.86	0.01	0.2
**CXCL10/IP-10**	LDH	11	0.69	0.02	0.25
**IL-6**	Procalcitonin	15	0.6	0.02	0.25
**CXCL9/MIG**	Creatinine	51	0.33	0.02	0.25
**Eotaxin**	D-Dimer	6	-0.88	0.02	0.26
**TNF-β**	LDH	11	-0.68	0.02	0.26
**CSF-1/M-CSF**	Platelet	51	-0.32	0.02	0.26
**IL-9**	C-Reactive Protein	7	-0.82	0.02	0.26
**IL-27**	Platelet	51	-0.31	0.03	0.29
**IL-8**	Creatinine	51	0.3	0.03	0.29
**IL-13**	C-Reactive Protein	7	-0.8	0.03	0.29
**IL-17A**	Ferritin	7	-0.8	0.03	0.29
**IL-17F**	Ferritin	7	-0.8	0.03	0.29
**IL-13**	Procalcitonin	15	-0.55	0.04	0.32
**TNF-α**	Creatinine	51	0.29	0.04	0.32
**IL-1RA**	Creatinine	51	0.29	0.04	0.35
**G-CSF**	Platelet	51	-0.28	0.04	0.37
**G-CSF**	Creatinine	51	0.28	0.05	0.4
**CCL2/MCP1**	Procalcitonin	15	0.52	0.05	0.4
**IL-6**	Platelet	51	-0.28	0.05	0.4

b. Among COVID-19 patients

Cytokine	Lab	N	R	p-value	q-value

**PDGF-AB/PDGF-BB**	Platelet	85	0.71	<.001	**<.001**
**PDGF-AA**	Platelet	85	0.66	<.001	**<.001**
**IL-6**	C-Reactive Protein	50	0.75	<.001	**<.001**
**CXCL10/IP-10**	C-Reactive Protein	50	0.6	<.001	**<.001**
**TNF-α**	C-Reactive Protein	50	0.59	<.001	**<.001**
**IL-7**	Platelet	85	0.45	<.001	**<.001**
**IL-6**	LDH	51	0.56	<.001	**<.001**
**CXCL9/MIG**	Ferritin	42	0.59	<.001	**0.002**
**Fractalkine**	Creatinine	84	0.42	<.001	**0.003**
**EGF**	Platelet	85	0.41	<.001	**0.003**
**CXCL9/MIG**	Procalcitonin	73	0.44	<.001	**0.004**
**TNF-α**	Ferritin	42	0.54	<.001	**0.006**
**IL-12 (p40)**	Creatinine	84	0.37	<.001	**0.01**
**IL-6**	Procalcitonin	73	0.39	<.001	**0.02**
**CXCL9/MIG**	Creatinine	84	0.35	0.001	**0.03**
**IL-10**	C-Reactive Protein	50	0.43	0.002	**0.04**
**IL-27**	C-Reactive Protein	50	0.43	0.002	**0.04**
**IL-10**	Ferritin	42	0.45	0.003	0.06
**IL-27**	Procalcitonin	73	0.34	0.003	0.06
**FGF-2**	Ferritin	42	0.44	0.004	0.06
**IL-18**	Ferritin	42	0.44	0.004	0.06
**IL-4**	C-Reactive Protein	50	-0.4	0.004	0.07
**CXCL10/IP-10**	Procalcitonin	73	0.33	0.004	0.07
**FLT-3L**	C-Reactive Protein	50	0.39	0.005	0.07
**CSF-1/M-CSF**	Ferritin	42	0.42	0.006	0.08
**FGF-2**	C-Reactive Protein	50	0.39	0.006	0.08
**CXCL10/IP-10**	Ferritin	42	0.42	0.006	0.08
**FLT-3L**	Ferritin	42	0.41	0.006	0.08
**IFN-γ**	Ferritin	42	0.41	0.006	0.08
**IL-15**	Ferritin	42	0.41	0.007	0.08
**TNF-α**	Procalcitonin	73	0.31	0.008	0.1
**IL-17A**	Creatinine	84	0.28	0.009	0.1
**IL-8**	Ferritin	42	0.4	0.009	0.1
**IL-1RA**	C-Reactive Protein	50	0.36	0.009	0.1
**IL-1RA**	Ferritin	42	0.39	0.01	0.1
**IL-6**	Ferritin	42	0.39	0.01	0.1

**CSF-1/M-CSF**	Procalcitonin	73	0.29	0.01	0.11
**CCL2/MCP1**	D-Dimer	31	0.44	0.01	0.12
**EGF**	Procalcitonin	73	-0.29	0.01	0.12
**CCL2/MCP1**	Procalcitonin	73	0.28	0.02	0.14
**FGF-2**	Procalcitonin	73	0.28	0.02	0.14
**G-CSF**	C-Reactive Protein	50	0.34	0.02	0.14
**IL-15**	Creatinine	84	0.26	0.02	0.15
**CCL4/MIP-1β**	Ferritin	42	0.36	0.02	0.16
**CXCL10/IP-10**	LDH	51	0.32	0.02	0.17
**TNF-α**	LDH	51	0.32	0.02	0.17
**IL-18**	Procalcitonin	73	0.27	0.02	0.17
**CCL7/MCP3**	D-Dimer	31	0.4	0.02	0.17
**CCL2/MCP1**	LDH	51	0.32	0.02	0.17
**CSF-1/M-CSF**	C-Reactive Protein	50	0.32	0.02	0.17
**CCL22/MDC**	Procalcitonin	73	-0.26	0.03	0.19
**CCL2/MCP1**	Ferritin	42	0.33	0.03	0.21
**sCD40L**	C-Reactive Protein	50	0.3	0.03	0.21
**IL-22**	Procalcitonin	73	-0.25	0.03	0.21
**CCL7/MCP3**	Procalcitonin	73	0.25	0.03	0.21
**TNF-β**	Procalcitonin	73	0.24	0.04	0.24
**IL-17E/IL-25**	Ferritin	42	0.32	0.04	0.24
**IL-1β**	Creatinine	84	0.22	0.04	0.25
**CCL2/MCP1**	C-Reactive Protein	50	0.29	0.04	0.25
**TNF-β**	Ferritin	42	0.31	0.04	0.26
**IL-18**	C-Reactive Protein	50	0.28	0.05	0.27
**TGF-α**	Ferritin	42	0.31	0.05	0.27
**IL-10**	LDH	51	0.28	0.05	0.27

Supplementary Table 8. Associations between day 1 of hospital admission cytokine expression levels and clinical outcomes. Hazard ratios and 95% confidence intervals (CI) are estimated from Cox Proportional Hazard models with robust standard errors. P-values with and without (q-value) adjustment for multiple comparisons are shown.

a. Development of Acute Kidney Injury

Cytokine	Hazard Ratio for AKI (95% CI)	p-value	q-value
**FGF-2**	1.49 (1.21, 1.84)	<0.001	**<0.001**
**IL-1β**	1.42 (1.21, 1.67)	<0.001	**<0.001**
**IL-1RA**	1.47 (1.25, 1.73)	<0.001	**<0.001**
**IL-4**	1.85 (1.34, 2.55)	<0.001	**<0.001**

**IL-6**	1.33 (1.15, 1.54)	<0.001	**<0.001**
**IL-12 (p40)**	1.53 (1.24, 1.89)	<0.001	**<0.001**
**IL-13**	1.61 (1.33, 1.96)	<0.001	**<0.001**
**IL-15**	1.84 (1.39, 2.45)	<0.001	**<0.001**
**IL-17A**	1.4 (1.2, 1.63)	<0.001	**<0.001**
**CCL3/MIP-1α**	1.59 (1.29, 1.97)	<0.001	**<0.001**
**Fractalkine**	2.5 (1.4, 4.47)	0.002	**0.007**
**CSF-1/M-CSF**	1.79 (1.16, 2.75)	0.008	**0.02**
**TGF-α**	1.14 (1.04, 1.24)	0.005	**0.02**
**G-CSF**	1.29 (1.06, 1.58)	0.01	**0.04**
**TNF-α**	1.66 (1.09, 2.51)	0.02	**0.047**
**EGF**	0.71 (0.52, 0.97)	0.03	0.07
**CCL4/MIP-1β**	1.59 (0.99, 2.56)	0.06	0.13
**IL-18**	1.4 (0.96, 2.05)	0.08	0.17
**IL-9**	1.32 (0.96, 1.83)	0.09	0.18
**IL-17E/IL-25**	1.24 (0.96, 1.6)	0.1	0.2
**IL-10**	1.25 (0.93, 1.69)	0.14	0.25
**IFN-α2**	1.31 (0.89, 1.95)	0.18	0.31
**CXCL10/IP-10**	1.16 (0.92, 1.46)	0.2	0.34
**PDGF-AA**	0.84 (0.63, 1.11)	0.22	0.36
**IL-8**	1.21 (0.86, 1.71)	0.28	0.44
**IL-5**	1.22 (0.83, 1.81)	0.32	0.48
**PDGF-AB/PDGF-BB**	0.85 (0.6, 1.2)	0.35	0.49
**TNF-β**	1.18 (0.84, 1.66)	0.34	0.49
**CXCL9/MIG**	1.16 (0.84, 1.61)	0.37	0.5
**IFN-γ**	1.09 (0.85, 1.39)	0.48	0.61
**CCL2/MCP1**	1.15 (0.78, 1.69)	0.48	0.61
**Eotaxin**	1.14 (0.72, 1.79)	0.58	0.68
**IL-27**	1.08 (0.84, 1.38)	0.55	0.68
**IL-7**	0.92 (0.64, 1.33)	0.66	0.76
**FLT-3L**	1.11 (0.64, 1.92)	0.7	0.78
**CCL7/MCP3**	0.87 (0.41, 1.87)	0.72	0.78
**CXCL1/GROα**	1.04 (0.81, 1.33)	0.77	0.81
**sCD40L**	0.99 (0.82, 1.2)	0.95	0.95
**CCL22/MDC**	1.02 (0.67, 1.56)	0.93	0.95

b. Mortality

Cytokine	Hazard Ratio for Mortality (95% CI)	p-value	q-value
**IFN-α2**	2.18 (1.48, 3.21)	<0.001	**<0.001**
**IL-13**	2.11 (1.52, 2.92)	<0.001	**<0.001**
**TNF-β**	1.79 (1.35, 2.37)	<0.001	**<0.001**

**TGF-α**	1.27 (1.09, 1.49)	0.003	**0.03**
**IL-18**	0.76 (0.62, 0.92)	0.006	**0.049**
**IL-9**	1.53 (1.03, 2.28)	0.03	0.16
**IL-17E/IL-25**	1.53 (1.04, 2.24)	0.03	0.16
**CSF-1/M-CSF**	1.69 (1.03, 2.76)	0.04	0.16
**CCL22/MDC**	1.84 (1.06, 3.2)	0.03	0.16
**Eotaxin**	1.61 (0.99, 2.62)	0.06	0.2
**FLT-3L**	1.5 (1, 2.25)	0.05	0.2
**IL-8**	1.28 (0.98, 1.68)	0.07	0.24
**IL-5**	1.35 (0.96, 1.9)	0.08	0.25
**EGF**	1.56 (0.9, 2.69)	0.11	0.31
**Fractalkine**	1.47 (0.79, 2.72)	0.23	0.42
**G-CSF**	1.13 (0.94, 1.35)	0.18	0.42
**CXCL1/GROα**	1.19 (0.92, 1.55)	0.19	0.42
**IL-4**	1.56 (0.77, 3.17)	0.22	0.42
**IL-6**	0.9 (0.75, 1.08)	0.25	0.42
**IL-12 (p40)**	1.28 (0.87, 1.89)	0.21	0.42
**CCL2/MCP1**	1.34 (0.85, 2.1)	0.21	0.42
**CCL7/MCP3**	1.29 (0.85, 1.94)	0.23	0.42
**PDGF-AB/PDGF-BB**	1.21 (0.87, 1.69)	0.25	0.42
**IL-1β**	1.27 (0.82, 1.97)	0.29	0.47
**IFN-γ**	0.79 (0.49, 1.27)	0.34	0.52
**IL-17A**	1.25 (0.75, 2.07)	0.39	0.56
**IL-27**	0.89 (0.69, 1.16)	0.39	0.56
**CXCL9/MIG**	1.16 (0.82, 1.65)	0.4	0.56
**PDGF-AA**	1.12 (0.84, 1.49)	0.43	0.57
**TNF-α**	1.18 (0.76, 1.82)	0.46	0.6
**IL-1RA**	1.05 (0.87, 1.27)	0.61	0.71
**IL-10**	1.06 (0.83, 1.34)	0.64	0.71
**CXCL10/IP-10**	0.96 (0.82, 1.13)	0.63	0.71
**CCL3/MIP-1α**	1.21 (0.63, 2.29)	0.57	0.71
**CCL4/MIP-1β**	0.88 (0.53, 1.47)	0.63	0.71
**IL-7**	1.07 (0.73, 1.57)	0.72	0.78
**sCD40L**	0.98 (0.74, 1.31)	0.91	0.95
**FGF-2**	0.95 (0.36, 2.52)	0.92	0.95
**IL-15**	1.01 (0.3, 3.44)	0.98	0.98

c. Development of Acute Respiratory Distress Syndrome

**Cytokine**	**Hazard Ratio for ARDS (95% CI)**	**p-value**	**q-value**
**IL-1RA**	1.35 (1.19, 1.53)	<0.001	**<0.001**
**IL-6**	1.34 (1.18, 1.52)	<0.001	**<0.001**

**CXCL10/IP-10**	1.48 (1.32, 1.67)	<0.001	**<0.001**
**TNF-α**	2.03 (1.46, 2.8)	<0.001	**<0.001**
**CCL7/MCP3**	2.56 (1.56, 4.22)	<0.001	**0.002**
**G-CSF**	1.2 (1.08, 1.34)	<0.001	**0.004**
**IL-13**	1.29 (1.11, 1.49)	<0.001	**0.004**
**CSF-1/M-CSF**	1.73 (1.25, 2.39)	<0.001	**0.004**
**Fractalkine**	2.02 (1.31, 3.11)	0.001	**0.006**
**PDGF-AB/PDGF-BB**	0.76 (0.64, 0.9)	0.002	**0.007**
**IL-8**	1.45 (1.12, 1.87)	0.005	**0.02**
**CCL2/MCP1**	1.67 (1.17, 2.39)	0.005	**0.02**
**PDGF-AA**	0.78 (0.65, 0.94)	0.009	**0.03**
**TGF-α**	1.1 (1.02, 1.2)	0.02	0.06
**TNF-β**	1.26 (1.03, 1.54)	0.02	0.06
**IL-10**	1.23 (1.01, 1.5)	0.04	0.09
**FLT-3L**	1.31 (0.98, 1.75)	0.07	0.15
**IL-15**	1.3 (0.97, 1.75)	0.08	0.15
**IL-27**	1.23 (0.99, 1.53)	0.07	0.15
**sCD40L**	1.13 (0.97, 1.33)	0.13	0.25
**IL-9**	1.18 (0.94, 1.48)	0.15	0.26
**IL-18**	1.25 (0.93, 1.68)	0.15	0.26
**CCL4/MIP-1β**	1.3 (0.92, 1.84)	0.14	0.26
**Eotaxin**	1.29 (0.89, 1.87)	0.18	0.27
**FGF-2**	1.19 (0.92, 1.55)	0.18	0.27
**CCL22/MDC**	0.81 (0.61, 1.09)	0.16	0.27
**CXCL1/GROα**	1.12 (0.92, 1.36)	0.27	0.39
**IL-17E/IL-25**	1.11 (0.9, 1.37)	0.31	0.43
**EGF**	0.86 (0.63, 1.18)	0.35	0.47
**IL-1β**	1.09 (0.89, 1.33)	0.4	0.52
**IL-12 (p40)**	1.09 (0.88, 1.35)	0.44	0.53
**CXCL9/MIG**	1.13 (0.84, 1.5)	0.42	0.53
**IL-4**	0.82 (0.43, 1.56)	0.55	0.65
**IL-5**	1.08 (0.84, 1.38)	0.57	0.65
**IFN-γ**	1.03 (0.88, 1.21)	0.69	0.77
**IL-17A**	1.03 (0.82, 1.3)	0.8	0.86
**IFN-α2**	1.02 (0.76, 1.38)	0.9	0.95
**IL-7**	0.99 (0.76, 1.3)	0.96	0.96
**CCL3/MIP-1α**	1.01 (0.81, 1.25)	0.95	0.96

Supplementary Table 9. Baseline demographics of Autopsy patients

Characteristic	COVID-19 Positive N=9	Lung controls N=	Kidney controls N=5
**Age**	67.4		57.8
**Sex, Female**	22% (2)		60% (3)
**Race**			
**Asian**	2		
**Black**	2		2
**Not Specified**	0		2
**Other(Hispanic)**	2		
**White**	3		1
**BMI**	27.75 (n=8)		30.35 (n=2)
**Smoking status**	4 former, no current		unknown
**Underlying lung disease**	2 OSA, 1 COPD		1 COPD, 1 Bronchitis
**Underlying kidney disease**	1 ESRD (DN)		none

Supplementary Figure 1. Cytokine expression of COVID-19 and control patients

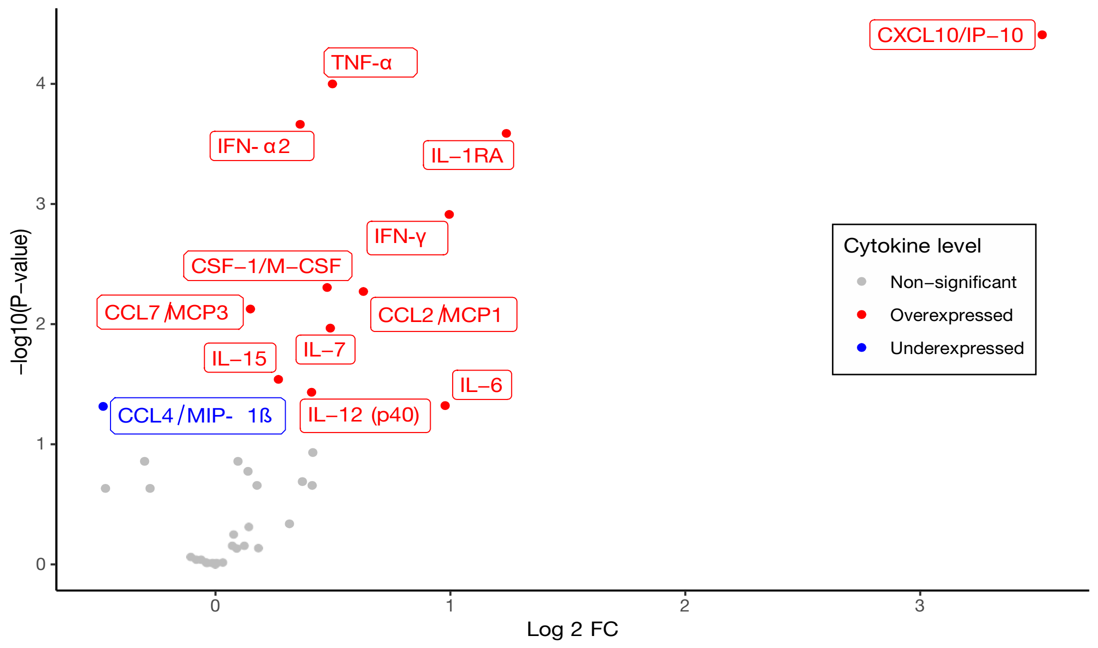

Volcano plot showing the log fold-change expression of significant cytokines between COVID-19 patients versus controls. P-values are on the negative log 10 scale and were computed from linear regression models with robust standard errors, after adjustment for multiple comparisons.

Supplementary Figure 2. Cytokine trajectories of COVID-19 patients with repetitive measures.

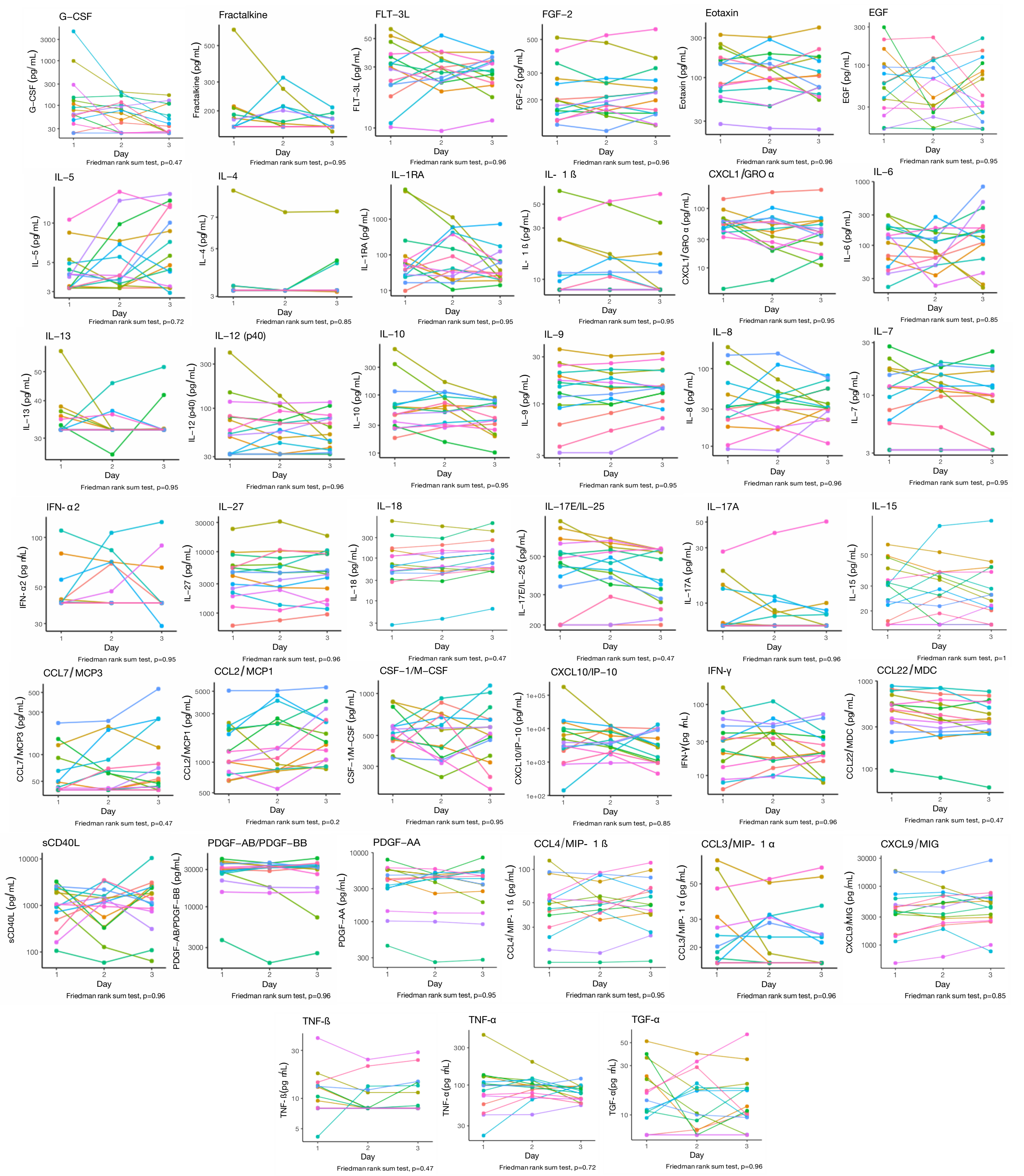

Cytokine levels of COVID-19 patients with samples at day 1, 2, and 3 of admission to the general wards. Each line represents a subject (COVID-19 patients with repetitive measures, n=15).

Supplementary Figure 3. Cytokine expression levels and SOFA scores in COVID-19 and control patients.

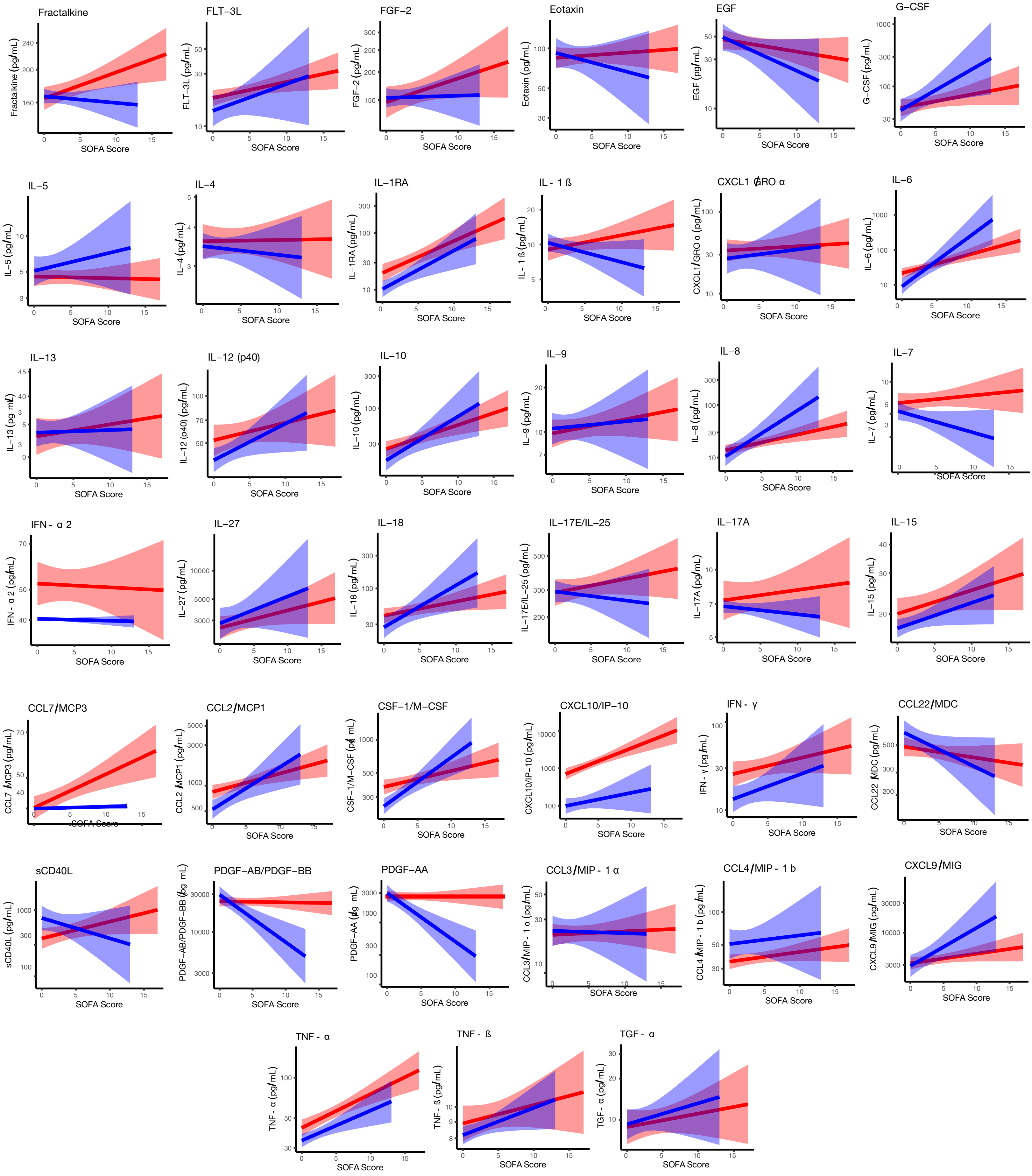

Blue indicates control patients and red indicates COVID-19 patients. The shaded area represents a 95% confidence interval around a line of best fit through the data points.

Supplementary Figure 4. Acute Kidney Injury staging and cytokine expression level correlations in COVID-19 positive patients.

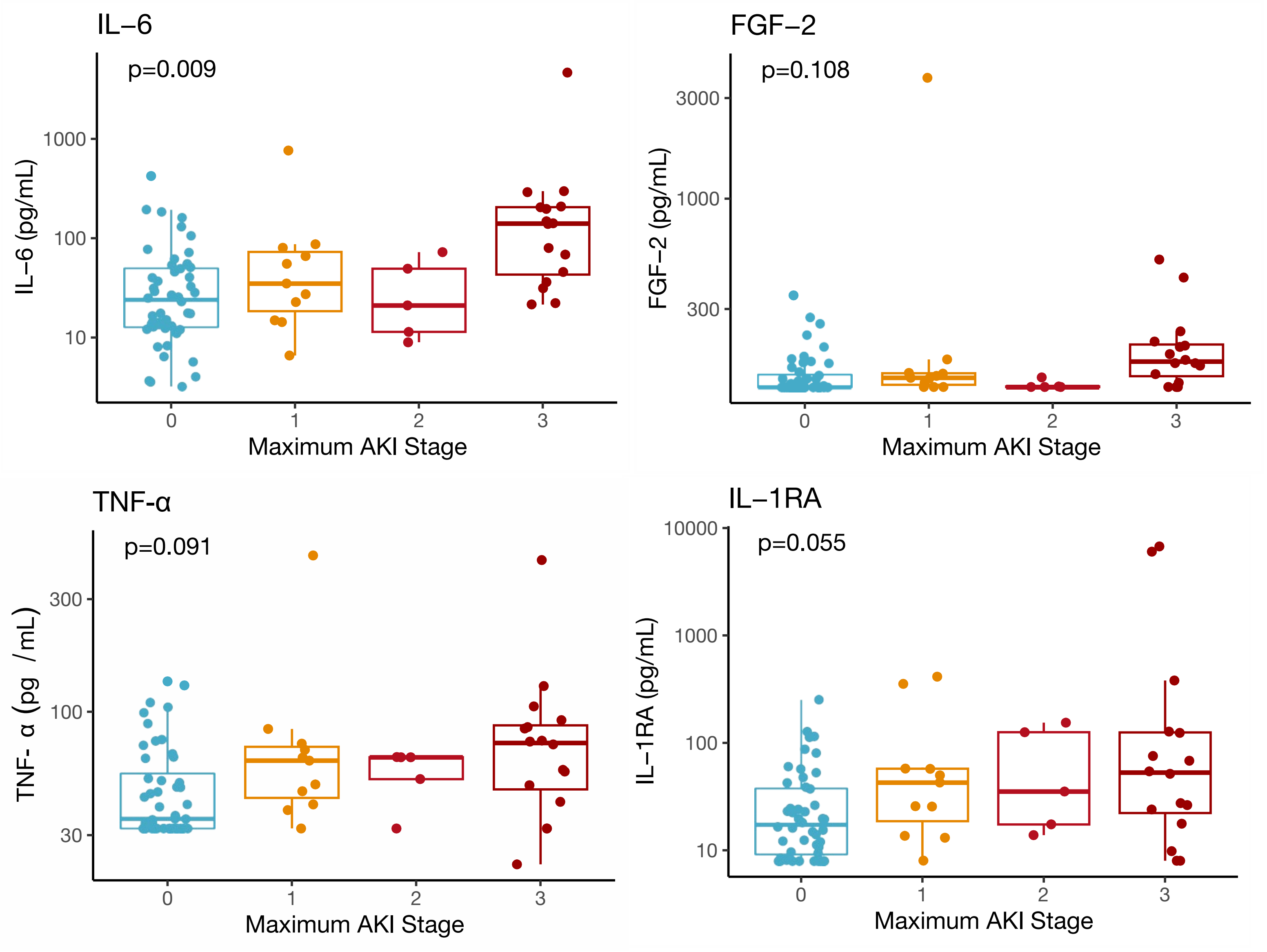

The horizontal line in the box plot represents the median and interquartile range. Each dot represents a subject. P-values accompanying each comparison were computed from Spearman's correlation formula and adjusted for multiple comparison.
